# Phosphorylation at the Homotypic Interface Regulates Nucleoprotein Oligomerization and Assembly of the Influenza Virus Replication Machinery

**DOI:** 10.1371/journal.ppat.1004826

**Published:** 2015-04-13

**Authors:** Arindam Mondal, Gregory K. Potts, Anthony R. Dawson, Joshua J. Coon, Andrew Mehle

**Affiliations:** 1 Medical Microbiology and Immunology, University of Wisconsin, Madison, Wisconsin, United States of America; 2 Department of Chemistry, University of Wisconsin, Madison, Wisconsin, United States of America; 3 Cellular and Molecular Biology Graduate Program, University of Wisconsin, Madison, Wisconsin, United States of America; 4 Department of Biomolecular Chemistry, University of Wisconsin, Madison, Wisconsin, United States of America; Virginia-Maryland Regional College of Veterinary Medicine, UNITED STATES

## Abstract

Negative-sense RNA viruses assemble large ribonucleoprotein (RNP) complexes that direct replication and transcription of the viral genome. Influenza virus RNPs contain the polymerase, genomic RNA and multiple copies of nucleoprotein (NP). During RNP assembly, monomeric NP oligomerizes along the length of the genomic RNA. Regulated assembly of the RNP is essential for virus replication, but how NP is maintained as a monomer that subsequently oligomerizes to form RNPs is poorly understood. Here we elucidate a mechanism whereby NP phosphorylation regulates oligomerization. We identified new evolutionarily conserved phosphorylation sites on NP and demonstrated that phosphorylation of NP decreased formation of higher-order complexes. Two phosphorylation sites were located on opposite sides of the NP:NP interface. In both influenza A and B virus, mutating or mimicking phosphorylation at these residues blocked homotypic interactions and drove NP towards a monomeric form. Highlighting the central role of this process during infection, these mutations impaired RNP formation, polymerase activity and virus replication. Thus, dynamic phosphorylation of NP regulates RNP assembly and modulates progression through the viral life cycle.

## Author Summary

Replication and transcription by negative-sense RNA viruses occurs in large macromolecular complexes. These complexes contain the viral polymerase, genomic RNA, and multiple copies of nucleoprotein that bind RNA and oligomerize to coat the genome. For influenza virus, nucleoprotein (NP) non-specifically binds nucleic acids and spontaneously oligomerizes. It is essential that a portion of NP be maintained as a monomer so that it can selectively oligomerize into replication complexes. Despite the fact that this process must be tightly regulated during the viral life cycle, how this regulation is achieved is largely unknown. Here we show that phosphorylation of NP negatively regulates assembly of the influenza virus replication machinery. We identified two phosphorylation sites on opposite sides of the NP:NP interface and showed that phosphorylation at either site blocks homotypic interactions, distorting the monomer:oligomer balance of NP in cells and severely impairing virus replication. Our findings show that the phospho-regulated conversion of NP between mono- and oligomeric states is important for RNP formation, gene expression and viral replication. Moreover, we showed that these critical phosphorylation sites play the same role in influenza B virus and are likely present in influenza C and D viruses, suggesting our results are broadly applicable across viral strains and genera and reveal a global regulatory strategy for *Orthomyxoviridae*.

## Introduction

Influenza viruses are major human respiratory pathogens that cause isolated seasonal outbreaks as well as the sporadic emergence of severe pandemics [1]. Influenza A virus (*Orthomyxoviridae*) is a segmented, negative-sense RNA virus. Like all other negative-sense RNA viruses, the influenza virus genome associates with the viral RNA-dependent RNA polymerase and multiple copies of the viral nucleoproteins (NP) to form ribonucleoprotein complexes (RNP) [2]. Within the RNP, the hetero-trimeric polymerase (composed of subunits PB1, PB2 and PA) catalyzes both transcription of viral messages and replication of the viral genome using an RNA template that is encapsidated by oligomeric NP [2, 3]. The regulated oligomerization of NP and assembly of the RNP is absolutely essential for successful infection, yet how influenza virus controls the formation of these multi-subunit complexes is largely unknown.

Upon infection, viral RNPs are released in to the host cell cytoplasm and actively transported to the nucleus [4]. In the nucleus, incoming RNPs are transcribed by their resident polymerase through a cap-snatching mechanism utilizing short host-derived 7mG-capped RNAs to prime synthesis of influenza mRNAs [5]. Synthesis of new proteins from the resultant mRNAs enables replication of the viral genome. Replication is primer independent and proposed to be performed in *trans* by a “free” RNA polymerase that copies the vRNP template to create positive-sense complementary-RNA (cRNA) [6]. cRNA synthesis is accompanied by concomitant encapsidation by NP to form cRNP complexes [7]. These cRNPs then direct the synthesis of new viral RNAs (vRNAs) and vRNPs that may be transcribed, template further replication, or be packaged into progeny virions.

cRNPs and vRNPs assume double helical structures composed of repeating NP subunits coating the genomic RNA [8–12]. Both genomic termini are located at the same end of the RNP where they are bound by the viral polymerase. This structural organization can be attributed to extensive intermolecular contacts between individual molecules of NP and between NP and genomic RNA. NP oligomerization occurs via a small “tail loop” (aa402-428) that projects away from the body of the protein and inserts into the binding groove of the interacting protomer [13, 14]. Multiple interactions between the tail loop and binding groove, notably a critical salt bridge between R416 of the tail loop with the E339 of the groove, contribute to self-association of NP and its ability to support RNP formation [13–18]. Additional contacts outside of the tail loop:binding groove interface are also important for the formation of higher-order structures; NP makes secondary intersubunit contacts thought to be important for helical strand formation, binds RNA via a patch of conserved basic residues located opposite the tail loop, and associates with the polymerase via conserved residues in a surface loop [9, 14, 19, 20].

Current models suggest that an NP monomer is initially recruited to form RNPs through direct interaction with the viral polymerase and binds to the nascent 5’ end of the viral genome [7]. This nucleates RNP assembly which is followed by NP:NP homo-oligomerization [8, 9, 13, 21–23]. The NP tail loop likely undergoes a conformational change during oligomerization, where it extends away from the body of the protein and fully exposes the RNA-binding surface [24]. RNA binding stabilizes the replication intermediates, and RNA binding and oligomerization are likely cooperative [24–26]. Incoming NP molecules unidirectionally extend the oligomer by inserting their tail loop into the binding groove of a pre-existing complex [14, 24, 25, 27].

When expressed alone, influenza NP self-assembles into oligomers and binds cellular RNA in a sequence-independent fashion [13, 14, 20, 24, 28]. Yet, RNP assembly during infection requires a fraction of NP to be maintained in an RNA-free monomeric form prior to its assembly into *bona fide* viral RNP complexes [24, 29]. Small molecules that disrupt this balance, by either inhibiting or forcing premature oligomerization, prevent RNP assembly and inhibit virus replication [16, 30–32]. Thus, in infected cells a regulated process must maintain a pool of NP monomers and simultaneously allow for the ordered assembly of new RNPs. Non-segmented RNA viruses solve this problem by encoding the phosphoprotein P. P maintains nucleoprotein in an RNA-free monomeric form and chaperones its assembly into the RNP [33–36]. Segmented RNA viruses like influenza lack any analogous viral proteins. It is currently unknown how influenza virus maintains a pool of monomeric, RNA-free NP that then dynamically changes to a high-order oligomeric state and encapsidates genomic RNA in the RNP.

NP is phosphorylated during infection, with patterns changing throughout the infectious cycle and dependent on both the viral strain and the host cell [37–40]. Phosphorylation occurs primarily on serine residues, several of which have been recently mapped in a phosphoproteomic survey of influenza virus proteins [24, 37, 39–41]. It was proposed over 35 years ago that phosphorylation may regulate its function [39], but the mechanism (s) remained largely unknown. Here we show that phosphorylation of NP inhibits oligomerization in cells and define the molecular mechanisms by which this modification impairs RNP formation. We identified three serine residues at the NP:NP interface that regulate oligomerization and play a critical during viral replication. We show that two of these residues are phosphorylated during infection. One of our newly identified phosphorylation sites, NP S407, resides in the tail loop, whereas the previously identified phosphorylation site S165 is at the entrance to the binding groove. Thus we demonstrate that phosphorylation on either side of the binding interface blocks NP:NP interactions. An additional residue at the binding groove entrance, S486, contributes to regulated assembly by preventing hyper-oligomerization. Mutation of these evolutionarily conserved residues or introduction of phosphomimetics distorts the monomer:oligomer balance in cells and severely impairs polymerase activity and virus replication. We show that a similar regulatory mechanism controls influenza B virus NP, suggesting a common strategy used throughout influenza virus genera. Our findings show that the regulated conversion of NP between mono- and oligomeric states is important for RNP formation, gene expression and viral replication, and support a model by which dynamic phosphorylation of NP regulates the viral replication machinery by controlling NP oligomerization.

## Results

### Phosphorylation negatively regulates NP self-association

**Fig 1:**
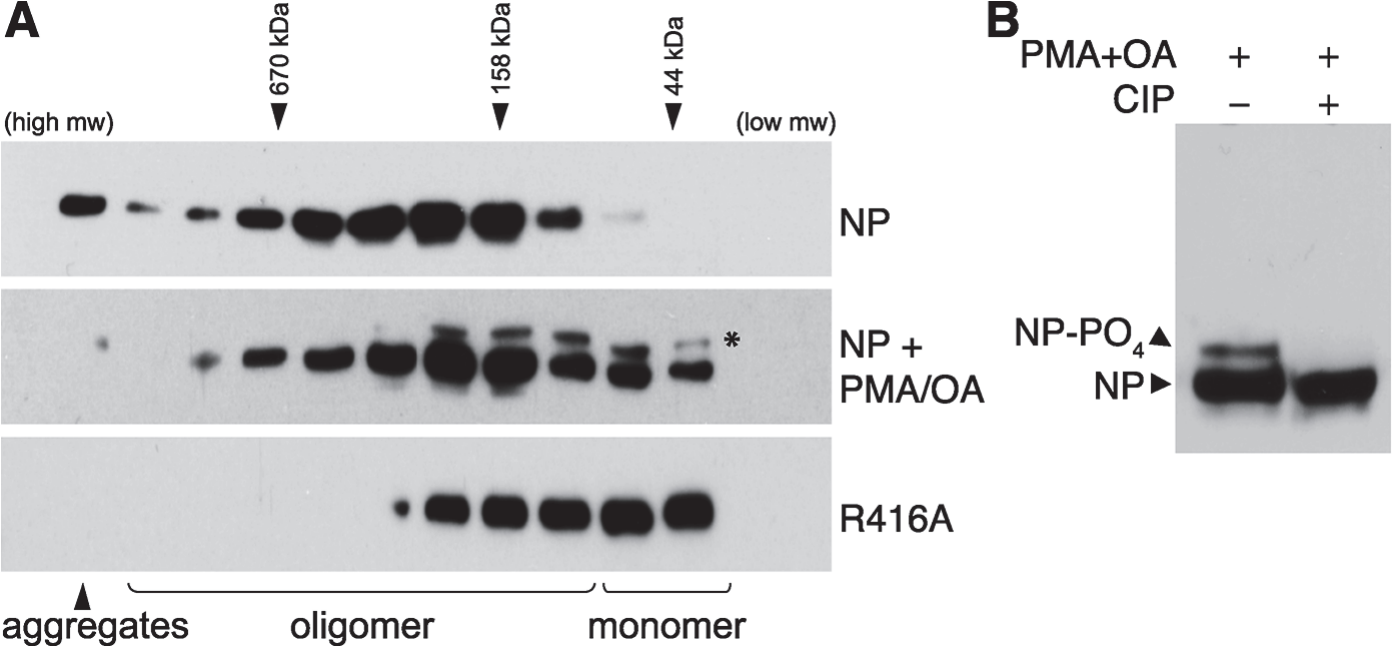
Phosphorylation disrupts self-association of NP. (A) The oligomerization state of influenza virus NP was determined by size exclusion chromatography. Human 293T cells were transfected with plasmids expressing either WT NP or the oligomerization-defective mutant NP R416A. To stimulate phosphorylation, cells were treated with PMA and okadaic acid (OA) or mock treated with DMSO. Whole cell extracts were treated with RNase, fractionated and analyzed by western blotting with anti-NP antibodies. * = stimulation induces the appearance of an NP species that migrates more slowly during SDS-PAGE. Elution peaks for calibration standards are shown. (B) PMA and OA induce NP phosphorylation. Lysates from stimulated cells expressing NP were treated with phosphatase (CIP) and analyzed by western blotting with anti-NP antibodies.

The mechanisms controlling NP oligomerization and RNP formation are poorly understood, although it has been recently suggested that post-translation modifications may be key regulators of this process [24, 41, 42]. We sought to examine whether phosphorylation of NP can modulate its oligomerization state in cells. Using the prototypical influenza strain A/WSN/33 as a model, the oligomerization state of NP was assessed by expressing protein in human 293T cells and separating the lysate by size exclusion chromatography. Wild-type (WT) NP formed a wide distribution of different oligomeric species with only a very minor portion eluting in the monomeric fractions (Fig 1A). In contrast, the previously characterized oligomerization-defective mutant NP R416A shifted dramatically to lower molecular weight species and eluted with a large portion of the protein in the monomer fractions. These data argue that the large majority of NP in cells spontaneously oligomerizes and that only a very minor fraction exists as a monomer. These findings agree with previous observations [17] and establish a robust system to analyze NP oligomerization in cells.

Phorbol 12-myristate 13-acetate (PMA) stimulates the phosphorylation of NP, presumably by activating its target proteins, protein kinase C (PKC) family members, and their downstream effectors [37]. To determine the effect of NP phosphorylation upon its oligomerization, cells expressing NP were treated with PMA and the phosphatase inhibitor okadaic acid prior to lysis and fractionation. PMA treatment significantly shifted the distribution of NP towards the monomeric fractions, suggesting phosphorylation inhibits oligomerization. Moreover, a distinct species of NP migrating more slowly in the gel was detected in the PMA-treated samples, suggestive of a hyperphosphorylated form. Treatment of the cell extracts with phosphatase completely eliminated the slower migrating species (Fig 1B), confirming that PMA stimulation induces NP hyperphosphorylation resulting in slower migration. The hyperphosphorylated species of NP was enriched in the lower molecular weight complexes (Fig 1A), further confirming that phosphorylation negatively regulates NP oligomerization and shifts NP towards a monomeric state.

### NP is phosphorylated in the tail loop and this regulates polymerase activity and viral replication

**Fig 2:**
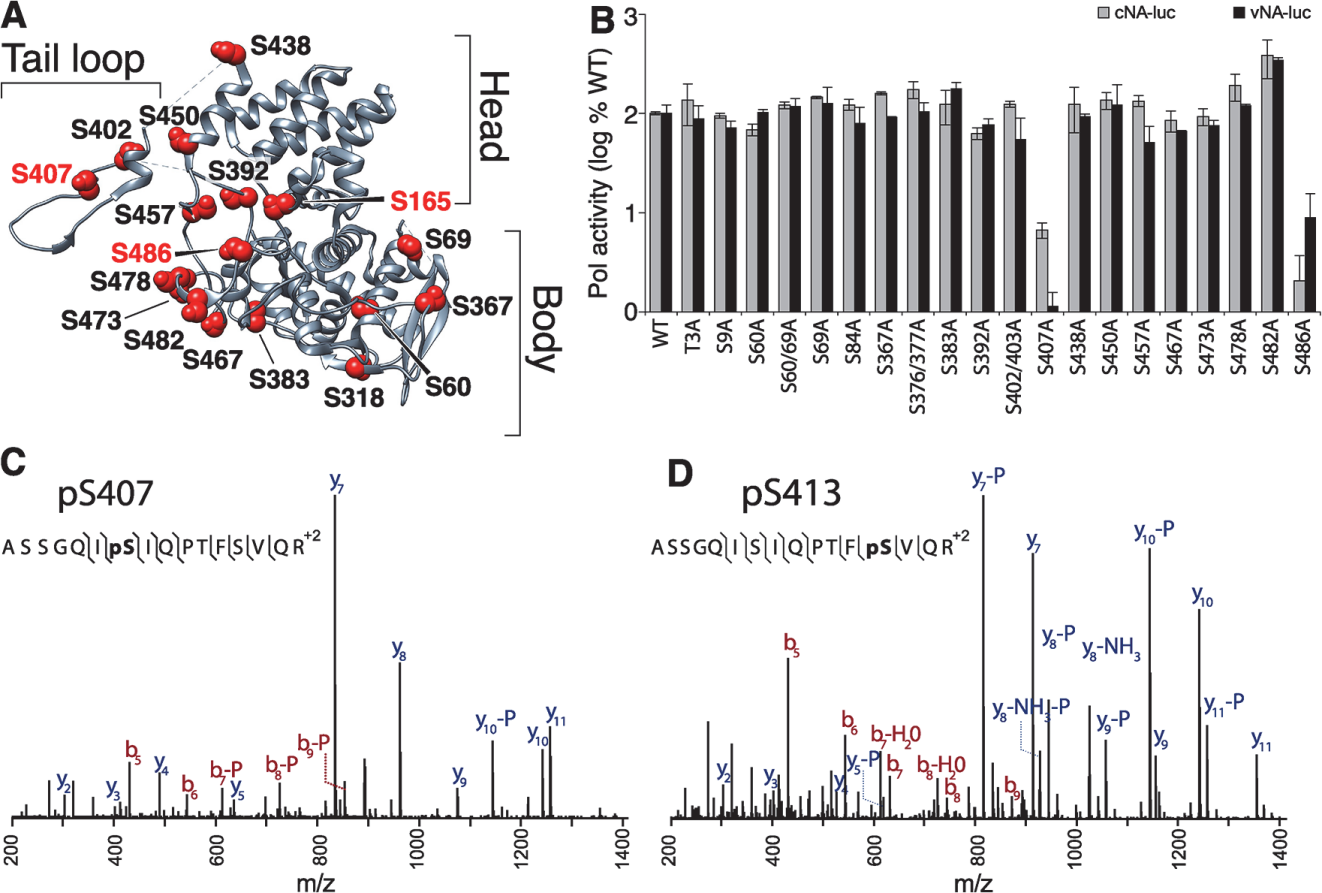
Identification of serine residues and a phosphorylation site in NP that are critical for polymerase activity. (A) The structure of NP (PDB 2IQH) reveals the core head and body regions as well as a tail loop that directs oligomerization. Conserved, solvent-exposed serine residues were identified in the NP protomer and selected for mutagenesis. (B) High-throughput polymerase activity assays were performed in human 293T cells expressing the viral polymerase, WT or mutant NP, and reporter constructs representing negative- (vNA-Luc) or positive-sense (cNA-Luc) RNA templates. (C, and D) Purified NP was prepared for mass spectrometry, tryptically digested, and enriched for phosphopeptides. Targeted MS identified the phosphopeptide 401-ASSGQISIQPTFSVQR-416 with phosphorylations localized to S407 and to S413 on NP.

Phospho-labeling has shown that most NP phosphorylation occurs on serine residues [37]. We therefore exploited an unbiased approach to identify serines that are important for polymerase function. Using the NP structure [13, 14], prior phospho-peptide analyses [43], and sequence conservation as a guide, we identified and mutated to alanine 20 surface-exposed serines that could potentially be phosphorylated (Fig 2A). Mutated serines were located in all three major structural domains—the head, body, and tail loop—and included residues identified by phosphoproteomics (i.e. S9, S402/403, S457 and S473). We also mutated the previously identified phosphorylation site at position 3 [43], which in the A/WSN/33 strain is a threonine.

High-throughput polymerase activity assays were performed in human cells expressing NP, the viral polymerase proteins PB1, PB2 and PA, and a vRNA-like reporter. To ensure sensitivity to minor changes in function, NP was expressed at limiting concentrations. Most NP mutants displayed activity within 2-fold of WT. Many of these mutations removed previously identified phosphorylation sites, indicating that not all phosphorylation sites in NP are essential for viral gene expression (Fig 2A and [41, 43, 44]). Strikingly, mutation at S407 and S486 drastically reduced polymerase function supporting less than 10% of the activity of WT NP. To determine if any of the NP mutants selectively block replication versus transcription, we repeated these assays using a cRNA-like reporter that requires at least one round of replication before it can serve as a template for mRNA production (Fig 2B). The NP S407A and S486A mutants continued to demonstrate severe defects in polymerase activity. However, no additional defects in NP function were revealed as all of the other mutants retained activity within 2-fold of WT. Immunofluorescence also showed that mutation at S407 or S486 did not alter the dynamic subcellular localization of NP (S1 Fig). Similar to WT, both NP S407A and S486A localize to the nucleus at early time points and redistribute to the cytoplasm at later time points. These data identify serine residues 407 and 486 in NP as important for supporting polymerase function.

Our findings motivated a focused analysis of NP phosphorylation to determine if S407 and/or S486 are post-translationally modified. NP was purified from infected cells and subject to high resolution mass spectrometry (S2A Fig and S1–S5 Tables). From this work we identified two new phosphorylation sites in the NP tail loop at S407 (Fig 2C) and S413 (Fig 2D). Two additional phosphorylation sites, S402, S403 were also identified in this experiment (S2B and S2C Fig). Phosphorylation had previously been partially localized to the tandem S402/S403 in the WSN strain [37, 41], but here we uniquely identify both of these residues as phosphorylation sites. These data show that tail loop is subject to multiple phosphorylation events. Interestingly, the NP S402A/S403A mutant exhibited only minor changes in polymerase activity (Fig 2B), suggesting that phosphorylation at this position is not essential for NP activity. As expected, phosphorylation was also identified at NP S165 (S2D Fig), a phosphorylation site that has been described previously and is important for polymerase activity [24, 41]. Despite identifying peptides containing residue S486, phosphorylation was not detected at this position.

**Fig 3:**
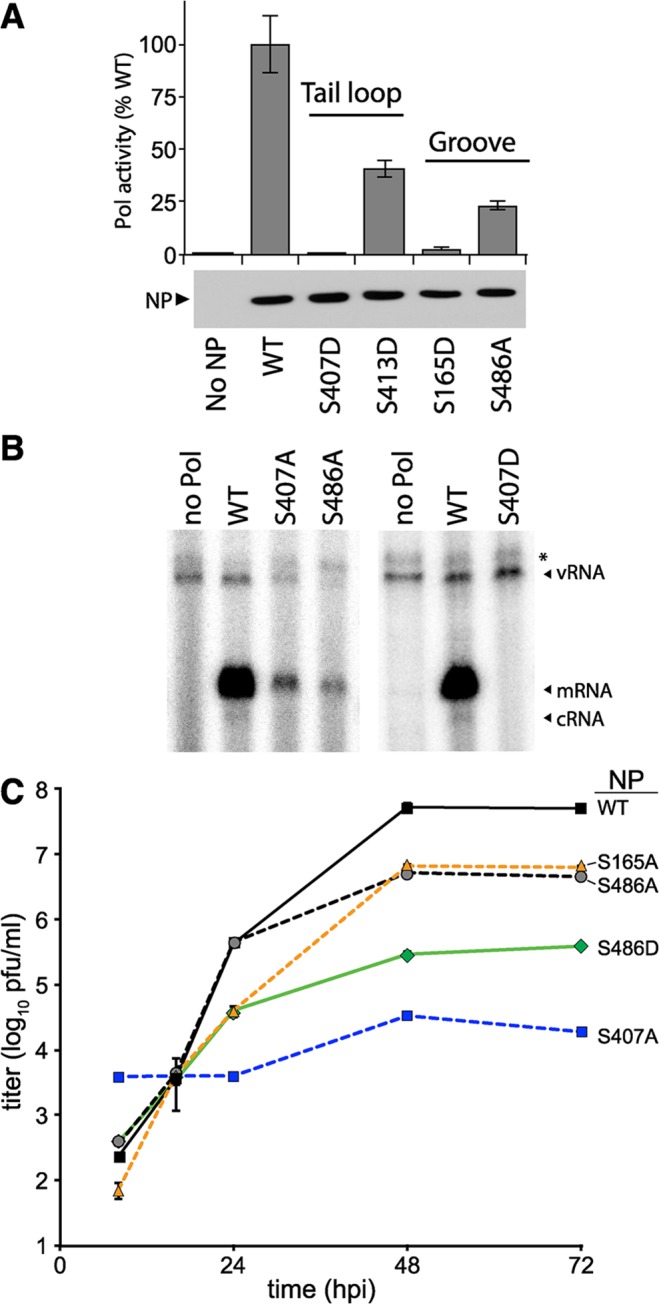
NP phosphorylation sites are important for RNP activity and virus replication. (A) Polymerase activity assay were performed with a vNA-luc reporter in the presence of excess WT or mutant NP. Equivalent expression of the wild type and mutant proteins were confirmed by western blotting cell lysates. Polymerase activity was normalized to that of WT (n = 3 +/- SD). (B) Primer-extension analysis was performed on RNA extracted from cells expressing the viral polymerase, vNA-luc, and WT or mutant NP. Polymerase products were identified by their predicted molecular weight. * = non-specific band. (C) Multicycle replication kinetics were examined in MDCK cells infected at an MOI of 0.01 with recombinant viruses encoding WT or mutant NP. Viral titers at the indicated time points were determined by plaque assay (n = 3 independent infections +/- SD).

To determine if these phosphorylations at the NP:NP interface are important for polymerase activity, we created phosphomimetic mutants and tested their functionality in polymerase activity assays where NP was expressed in excess, to best mimic conditions during infections. NP S407D and S413D mimic tail loop phosphorylations, while S165D mimics phosphorylation in the binding groove where the tail loop from the neighboring protomer interacts. Polymerase activity was severely impaired by the tail loop mutant NP S407D and was indistinguishable from background levels obtained in the absence of NP (Fig 3A). This was an additional ~10-fold decrease in activity compared to NP S407A. Similarly, the binding groove mutant S165D dramatically reduced polymerase activity. By contrast, NP S413D exhibited modest defects reducing polymerase activity by only ~50%, indicating that S413 is not an essential sites, and this position was not analyzed further. Confirming earlier results, even with higher levels of NP the S486A mutant at the entrance to the binding groove supported polymerase activity at only ~20% of the WT level. Western blotting confirmed comparable expression of WT and mutant NPs (Fig 3A).

Primer extension assays were performed to directly monitor production of viral RNAs in the presence of the NP mutants identified in our screen. Influenza polymerase in the presence of WT NP produced large amounts of viral mRNA in addition to low levels of the replication intermediate cRNA (Figs 3B and S3). vRNA was exogenously expressed and present in all conditions, increasing slightly in the presence of WT NP compared to conditions where the polymerase was absent. The NP mutants S407A, S486A, and S407D demonstrated decreased or undetectable levels of mRNA, cRNA, and vRNA when compared to WT NP. Compared to WT NP, the viral polymerase produced only 20–30% the amount of mRNA and background levels of cRNA in the presence of NP S407A or S486A (S3 Fig). In agreement with our polymerase activity assays, NP S407D exhibited the strongest defect with activity completely ablated. These mutants disrupted both gene transcription and genome replication, suggesting a defect in the early stages of RNP assembly.

Recombinant influenza virus was generated to test the impact of mutations at S486 and the phosphorylation sites S165 and S407 in the biologically relevant context of a viral infection. Multicycle replication assays were performed by infecting cells with virus encoding WT or mutant NP. Virus encoding NP S165A or S486A replicated to 10-fold lower levels than WT. NP S407A was the most severely attenuated, demonstrating a ~1000-fold decrease in viral titers compared to WT from 24–72 hours post-infection (hpi). Our results differ from those reported with A/Victoria/3/1975 strain, where NP S407A was functional in polymerase activity assays, although this was not tested with a recombinant virus [45]. NP S407A has also been suggested to possess a temperature sensitive phenotype [46]. Whether these different properties attributed to NP S407 arise from differences in experimental systems and viral strains, or possibly represent the presence of a redundant regulatory mechanism remains to be determined. The strength of the replication defect in our system for S407A and S486A mutants paralleled results from the polymerase activity assay. Despite multiple attempts, we were unable to rescue viruses encoding NP S407D, even when complementing with WT NP in *trans*, indicating an extreme defect caused by mimicking constitutive phosphorylation at this site. To further examine the function of S486, we tested the hypothetical scenario of S486 phosphorylation by assessing replication of the phosphomimetic NP S486D. Although phosphorylation was not detected at NP S486 (Fig 2 and [41]), this residue is located at the entrance to the binding groove opposite the phosphorylation site S165 and if it were phosphorylated it might impact engagement of the tail loop from incoming NP. The NP S486D mutation resulted in an intermediate phenotype, reducing replication an additional ~10-fold when compared to NP S486A (and ~100-fold when compared to WT), but was not as defective as NP S407A. Thus, our high-throughput polymerase activity assay identified conserved serine residues in NP that are important for high-level virus replication, including at least one novel phosphorylation site.

### Phosphorylation on either side of the NP:NP interface prevents multimerization

**Fig 4:**
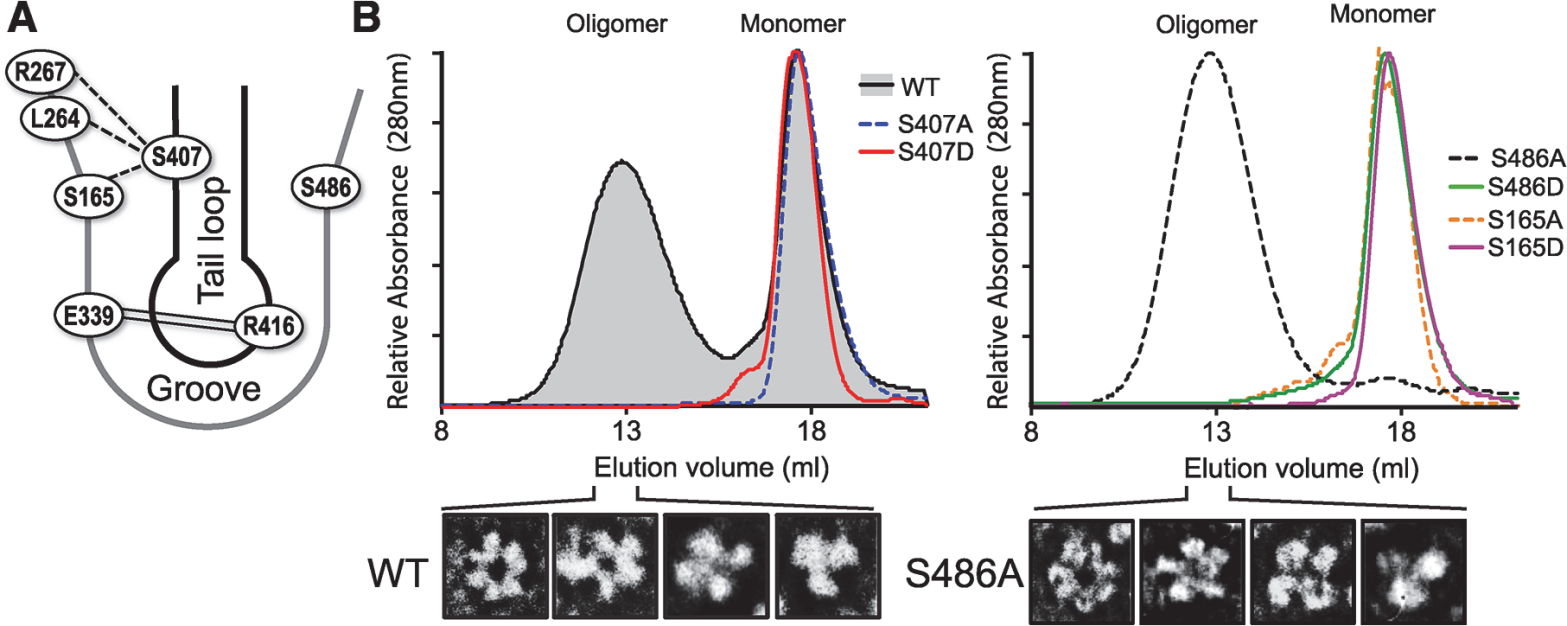
S407 and S486 are located at the NP:NP interface and influence oligomerization. (A) Schematic representation of the NP:NP interface highlighting the organization of S407 in the tail loop of one protomer and S486 and S165 in the binding groove of the binding partner. The salt bridge interaction between R416 and E339 is shown by double line. The crystal structure of NP suggests potential intermolecular H-bonding between S407 of the tail loop and residues S165, S264 and S267 in the groove, indicated by dotted lines [13]. (B) RNA-free WT, mutant, and phosphomimetic NP were purified from bacteria and analyzed by size exclusion chromatography. NP monomer and oligomer peaks are marked. Heterogeneity within the oligomeric WT and NP S486A populations was detected by negative stain electron microscopy, revealing NP trimers, tetramers, pentamers and hexamers.

Our data demonstrate that NP S165, S407, and S486 are important for viral replication (Fig 3). These residues are located at the NP:NP interface where the tail loop of one protomer inserts into the binding groove of the neighboring molecule, and S165 is known to be phosphorylated and important for oligomerization [13, 14, 41, 47, 48]. Combined with our data that NP S407 is phosphorylated (Fig 2C) and that phosphorylated NP favors a monomeric state (Fig 1), this immediately suggested that phosphorylation may interfere with oligomerization. Intersubunit interactions are dominated by hydrogen bonds and a critical salt bridge between R416 in the tail loop and E339 in the binding groove (Fig 4A) [13, 15]. Phosphorylation and/or mutation to alanine, which removes hydrogen bonding potential, is likely to significantly alter the local binding environment. The structure of NP suggests that S407 in the tail loop has the potential to participate in multiple hydrogen bonds with the binding groove, including with S165. Moreover, S486 is located at the entrance to the binding groove, opposite S165. In addition to disrupting hydrogen bonds important for oligomerization, the structures of NP suggest that a phosphate could not be accommodated at either S165 or S407 in the oligomer [13, 14]. To test these possibilities, we analyzed the oligomerization of recombinant, RNA-free protein. After extended incubation in solution to permit oligomerization to reach equilibrium [20], proteins were analyzed by size exclusion chromatography (Fig 4B). WT NP elutes as a mixture of monomeric and multimeric species, whereas the S165D mutation created an exclusively monomeric peak, as previously reported [20, 48]. The WT NP oligomer elutes as a broad peak, therefore to determine the exact oligomerization state individual fractions were analyzed by transmission electron microscopy. The recombinant NP oligomers are composed of different ring shaped molecules, ranging from trimers to hexamers (Figs 4B and S4). No such structures were observed in the monomer fraction.

NP mutants S165A and S407A eluted exclusively as monomers (Fig 4B), demonstrating that in the absence of RNA these serines are essential for oligomerization of recombinant NP, likely through H-bonding interactions between the tail loop and binding grove. The phosphomimetic NP S407D was also monomeric, suggesting that phosphorylation at this position may negatively regulate NP:NP assembly. By contrast, NP S486A showed the opposite effect, shifting the entire population to the oligomeric state. Transmission electron micrographs of the peak fraction showed that the S486A mutant forms ring-shaped structures ranging from trimer to hexamers, identical to WT NP (Figs 4B and S4). No monomeric peak was detected, suggesting that S486 is important in balancing the equilibrium of NP:NP interactions. NP S486 is located at the entrance to the binding groove opposite the known phosphorylation site S165, and NP S165D disrupts NP oligomerization. We asked whether a hypothetical phosphorylation at S486 on the other side of the binding groove might also disrupt oligomerization. Indeed, whereas NP S486A shifted the equilibrium completely to the oligomeric state, the phosphomimetic NP S486D was almost completely monomeric (Fig 4B).

**Fig 5:**
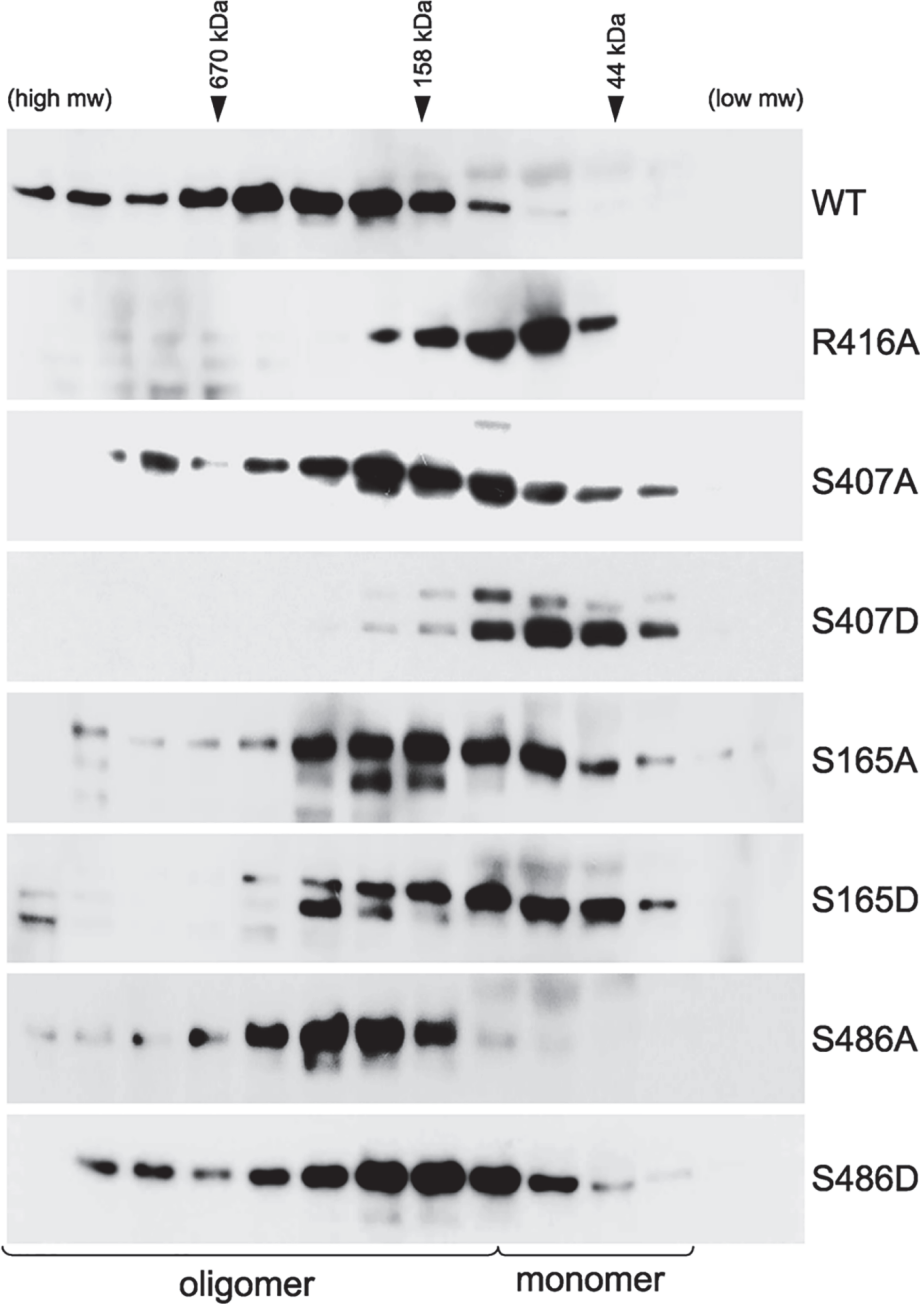
Critical serine residues control NP oligomerization in cells. Lysates were prepared from 293T cells expressing WT, mutant or phosphomimetic NP and fractionated by size exclusion chromatography. Fractions were analyzed by western blot with anti-NP antibodies. WT NP and the oligomerization defective NP R416A were used as internal standards to determine the position of the oligomeric and monomeric populations, respectively. Elution peaks for calibration standards are shown.

NP undergoes a number of post-translational modifications and interacts with several cellular factors, some of which have been proposed to modulate NP function[41, 49–51]. It is therefore important to assess NP oligomerization and its potential regulation by phosphorylation in eukaryotic cells. WT or mutant NPs were expressed in 293T cells and their self-association was assessed by size-exclusion chromatography after rigorous RNase treatment. Again, WT NP showed a characteristic distribution of different oligomeric forms and only a minor population of monomers, while the oligomerization mutant NP R416A eluted as a lower molecular weight species close to the expected position of a monomer (Fig 5). Paralleling results with recombinant protein, the mutants S407A and S407D drove NP towards a monomeric state, with the phosphomimetic NP S407D producing the most pronounced shift in oligomerization of all the mutants examined (Fig 5). Similarly, the NP mutants S165A and S165D also assumed a larger proportion of lower molecular weight complexes and the phosphomimetic mutant exhibited a more pronounced phenotype. NP S165A purified from cells possessed notably more multimers than the bacterially expressed RNA-free protein, in agreement with the high degree of oligomerization observed for NP S165A purified from insect cells [47]. Mutations at NP S486 induced an intermediate effect. As seen *in vitro*, NP S486A from cells eluted largely as an oligomer, although the distribution is more compact than the wild-type protein. Introducing NP S486D restored a more pronounced monomer population, but did not fully recapitulate the oligomerization defect of the recombinant protein. Our cell-based results thus reinforce those obtained with recombinant proteins. Together, these data identify new residues that make critical inter-subunit contacts during NP oligomerization and provide evidence that phosphorylation at the NP:NP interface directly regulates self-association.

### Phosphorylation disrupts tail loop-binding groove interactions essential for RNP assembly

**Fig 6:**
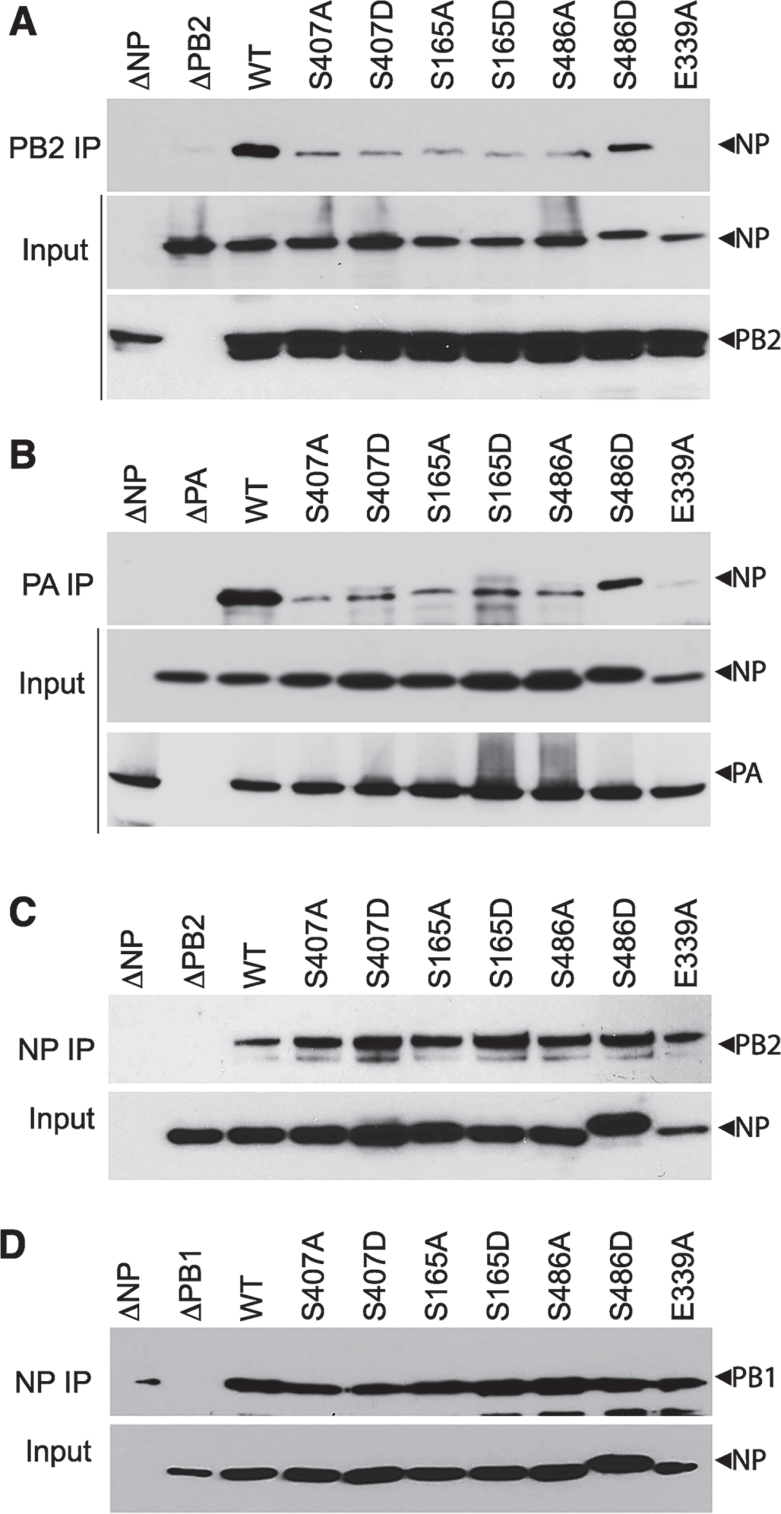
NP phospho-mutants disrupt RNP formation. (A and B) Viral RNPs were reconstituted in 293T cells by expressing WT or mutant NP-V5, a vRNA template, and polymerase containing either PB2-HA (A), or PA-FLAG (B). Assembled RNPs were immunoprecipitated using anti-HA or anti-FLAG antibodies and co-precipitated NP was visualized by blotting with anti-RNP antibody (upper panels, IP). Expression of PB2, PA and WT or mutant NP was confirmed by blotting whole cell lysate (lower panels, Input). (C and D) NP mutations do not disrupt binding to PB2 or PB1. Lysates were prepared from 293T cells expressing PB2-HA (C) or PB1-HA (D) and WT or mutant NP-V5, treated with RNaseA, and immunoprecipitated with anti-V5 antibody. Co-precipitating PB2 or PB1 was detected by blotting with anti-HA antibody (upper panels, NP IP). Equivalent expression of WT and mutant NP were confirmed by blotting whole cell extracts with anti-V5 antibody (lower panels, input).

We and others have shown that NP mutants with altered oligomerization profiles reduced polymerase activity and viral replication (Figs 2–5 and [16–18]). Each of these events is dependent upon successful formation of viral RNPs. To specifically investigate whether changes in NP self-association perturbs viral RNP formation in the presence of the viral polymerase and genomic RNA, NP mutants were used in an RNP reconstitution assay. In this assay viral RNPs were reconstituted in human cells by expressing the viral polymerase (PB2-HA, PB1 and PA), WT or mutant NP, and a vRNA-like template. The efficiency of RNP formation was determined by immunoprecipitating the viral polymerase via PB2-HA to isolate RNPs and detecting co-precipitated NP by western blotting. WT NP co-purified with the viral polymerase indicating efficient RNP formation (Fig 6A). As a control, the NP mutant E339A, which disrupts the critical inter-NP salt bridge [16], severely impaired RNP formation. Mutation of the phosphorylation sites NP S407 and S165 or introduction of phosphomimetics caused significant decreases in RNP formation, despite expression levels similar to WT. NP S486A showed a similar reduction in RNP formation, whereas S486D showed intermediate levels of RNP formation. Given that NP and free PB2 can interact directly and may result in co-precipitation independent of polymerase trimerization and RNP formation [52], we repeated these experiments isolating RNPs via PA-FLAG immunoprecipitation. PA and NP do not interact directly, therefore co-precipitation can only occur via interactions with the trimeric polymerase (Fig 6B). These experiments yielded identical results, where all of the NP mutants were severely impaired for RNP formation except for S486D that displayed an intermediate phenotype. These data analyzing RNP formation agree with the polymerase activity assays performed earlier that showed a significant loss of function for these mutants (Figs 2 and 3A). Notably, NP mutants that were primarily monomeric (e.g. S165D, S407D) or that were exclusively oligomeric (e.g. S486A) exhibited similar defects in RNP assembly.

To confirm that this defect in RNP formation is a result of the abnormal NP oligomerization and not due to any defects in NP-polymerase interactions, the binary interactions between NP and PB2 or PB1 were tested for all of the mutant proteins (Fig 6C and 6D). Lysates containing NP and either PB2 or PB1 were subject to NP immunoprecipitation and co-purification of the interacting partner was detected by western blot. To eliminate any non-specific complex formation containing cellular RNA, lysates were treated with high amounts of RNase A before immunoprecipitation. WT and mutant NP precipitated similar amounts of PB2 (Fig 6C) and PB1 (Fig 6D), suggesting that mutations at the NP:NP interface do not interfere with binding to the polymerase. Moreover, all of the NP mutants displayed proper subcellular localization, present in the nucleus early after expression and exported to the cytoplasm at later time points (S1 Fig). These data highlight the essential role that regulated NP oligomerization plays in RNP formation and raise the possibility that a balanced equilibrium between monomeric and oligomeric forms is crucial for RNP assembly and function.

**Fig 7:**
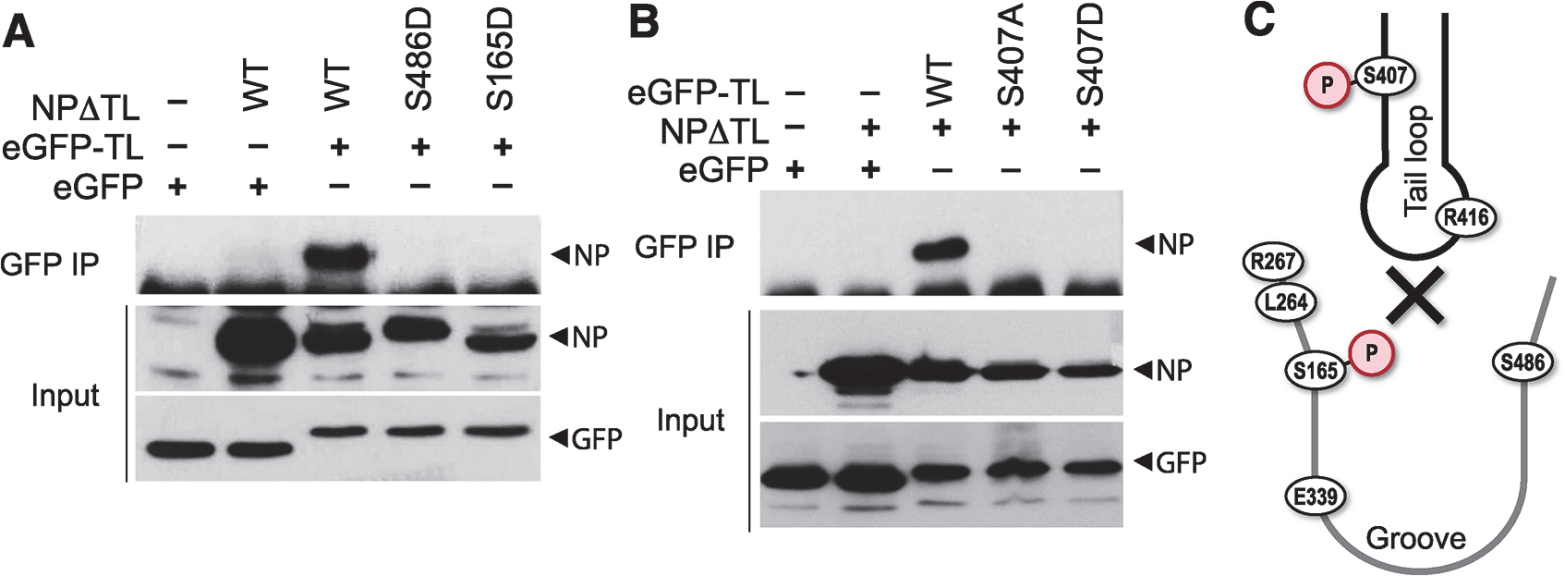
Phosphorylation of the tail loop and binding groove maintains monomeric NP. Binary tail loop-binding groove interactions were directly probed using an NP tail loop deletion mutant (NPΔTL) and a tail loop fused to eGFP (eGFP-TL). (A) Binding groove mutants were investigated by co-expressing WT or mutant NPΔTL with eGFP-TL in 293T cells. Lysates were immunoprecipitated with anti-GFP antibody and co-precipitating NP was visualized by blotting with anti-RNP antibody. Expression levels of interacting partners were analyzed by blotting total proteins with anti-RNP or anti-GFP antibodies. (B) The impact of tail loop mutants was probed by co-expressing WT or mutant eGFP-TL with NPΔTL. Complexes were immunoprecipitated and detected as above. (C) Schematic depiction of how phosphorylation might block entry of the tail loop into the binding groove and inhibiting NP oligomerization.

Based on our results, we hypothesized that phosphorylation at the NP:NP interface interferes with oligomerization through at least two possible mechanisms: 1) by modifying NP S407, thereby making the tail loop unsuitable for insertion into the existing oligomer, and 2) by modifying S165 and masking the binding groove to preclude accepting the tail loop from an incoming NP molecule. In both cases, phosphorylation would dynamically control NP:NP interactions and negatively regulate oligomerization and RNP formation. As NP can both be incorporated into a growing oligomer via its tail loop and subsequently accept a tail loop from the next incoming molecule, it is challenging to differentiate the exact oligomerization defect of our NP mutants using full-length protein. Therefore, we exploited a tail loop-binding groove interaction assay [16]. Binding assays were performed with a tail loop deletion mutant of NP (NPΔTL), which retains a functional binding groove but cannot self-associate due to the absence of the loop, and a GFP-tail loop fusion, which possesses only the tail loop (aa402-428). NPΔTL was co-precipitated by the GFP-tail loop fusion, but not with GFP alone, demonstrating a specific binary interaction between these two domains (Fig 7). NPΔTL was mutated to determine the impact of phosphorylation on binding groove functionality. The binding groove was completely defective upon introduction of the phosphomimetic residues S165D or S486D (Fig 7A). The GFP-tail loop protein was also investigated. The tail loop mutant S407A and the phosphomimetic S407D tail loop both failed to interact with NPΔTL (Fig 7B). All of the mutants were expressed equivalently to WT. These results suggest that phosphorylation at either side of the NP:NP interface blocks insertion of the tail loop into the binding groove and prevents NP oligomerization (Fig 7C). Furthermore, they reinforce our earlier findings demonstrating oligomerization defects for these mutants in the context of full-length proteins (Figs 4 and 5) and suggest that modifications at this interface alone can regulate NP oligomerization, and the downstream processes of RNP formation, gene expression and replication, and ultimately virion production.

### Phospho-regulation of NP oligomerization is conserved in influenza A and B viruses

**Fig 8:**
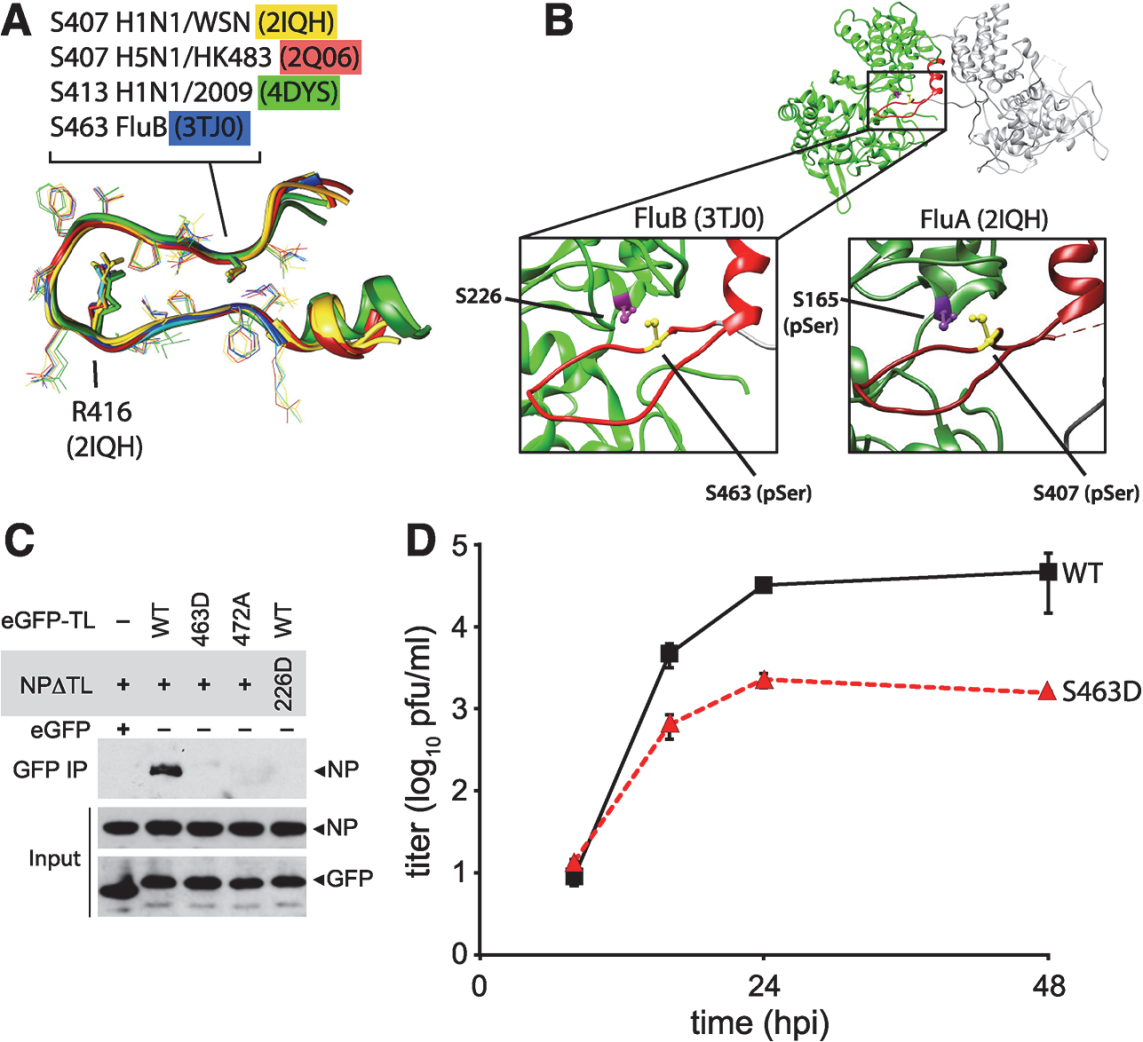
Phosphomimetic mutations in influenza B NP disrupt tail loop-binding groove interaction and attenuate virus replication. (A) Conserved tail loop conformations for influenza A and B virus NP. The phosphorylated NP S407 from influenza A NP is analogous to S463 in influenza B NP. NP derived from pandemic 2009 H1N1 (4DYS) contains a naturally occurring N-terminal addition, shifting the numbering scheme by six amino acids. (B) Interaction between individual protomers of influenza virus B NP. The tail loop is shown in red. For clarity, a cross-section of the structure is shown to reveal the tail-loop and binding groove. The spatial organization of S223 and S463 in influenza B NP is highlighted (left), revealing a nearly identical positioning to that of influenza A NP (right). (C) Tail loop:binding groove assay for influenza B NP using tail loop (R472A and S463D) and binding groove (S226D) mutants. Proteins were expressed in 293T cells and lysates were immunoprecipitated with anti-GFP antibody. Co-precipitating NP (upper panel) and proteins in the lysate (lower two panels) were analyzed by blotting with anti-V5 or anti-GFP antibodies. (D) Multicycle replication kinetics were examined in MDCK cells infected at an MOI of 0.01 with recombinant influenza B viruses encoding either WT or S463D mutant NP. Viral titers at the indicated time points were determined by plaque assay (n = 3 independent infections +/- SD).

Multiple structures of NP have been determined from diverse orthomyxoviruses, including influenza A virus [13, 14], influenza B virus [53], and even infectious salmon anemia virus [54]. While NP from different orthomyxoviruses displays limited sequence identity, the structures of each revealed a similar global architecture. Alignment of the tail loops from all of the influenza virus NP structures shows the structure of this region is completely conserved (Fig 8A). Furthermore, critical phosphorylation sites and salt bridge residues are retained at the same positions (Figs 8A and S5): the serine residue at the entrance to the binding grove in influenza A virus NP (S165) is present in influenza B virus NP (S226), and the critical features of the tail loop from influenza A NP (S407 and the salt-bridge residue R416) are also shared by influenza B NP (S463 and R472, respectively). In both influenza A and B NP structures, the serine residues in the tail loop and binding groove are apposed at the NP:NP interface (Fig 8B).

Phosphoproteomics of influenza B virus identified a phospho-peptide from the tail loop containing S463 [41]. We therefore used the tail loop-binding groove interaction assay to test whether oligomerization of influenza B NP (B/Brisbane/60/2008) is also regulated by phosphorylation. The tail loop of B NP was sufficient to mediate interactions with the binding groove, whereas mutation of the salt-bridge residue R472A disrupted binding (Fig 8C). Phosphomimetics in either the tail loop (S463D) or the binding groove (S226D) completely ablated binding. Recombinant influenza B viruses encoding either wild type of phosphomimetic NP were used to assess the impact of these mutations on virus replication. Introduction of the phosphomimetic S463D into the tail loop of influenza B NP attenuated multicycle virus replication by at least 100-fold with respect to the wild type (Fig 8D). Despite multiple attempts, we were unable to rescue virus encoding NP S226D, suggesting severe defects in function for this mutant. Thus, phosphorylation at the NP:NP interface controls homotypic binding and replication of influenza B virus, and phospho-regulation is a conserved mechanism modulating NP oligomerization for both influenza A and B viruses, and possibly other genera of *Orthomyxoviridae*.

## Discussion

**Fig 9:**
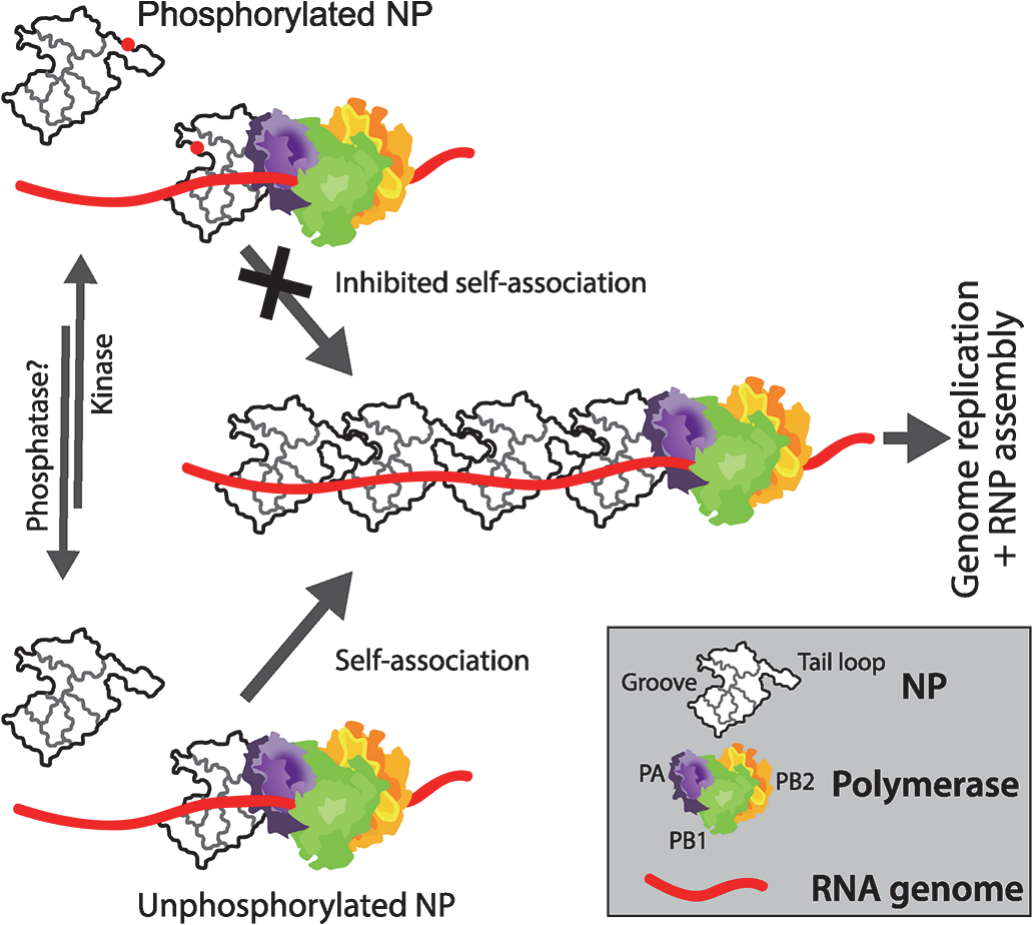
Mechanistic model for the regulated oligomerization of NP and RNP assembly. A portion of NP is phosphorylated by an unknown host kinase at S165 and S407 (represented as red dots), preventing tail loop:binding groove interactions and preventing self-association. Conversely, at the appropriate stage of the viral life cycle, dephosphorylation of NP by an unknown cellular phosphatase or synthesis of new NP molecules that do not get phosphorylated permits efficient self-association, genome replication, and RNP assembly.

The influenza virus RNP directs gene expression and genome replication. During assembly of the RNP, NP dynamically changes from the RNA-free monomeric state to the high-order oligomeric state that encapsidates genomic RNA in the RNP [20, 48]. NP oligomerizes by inserting a tail loop from one NP protomer into the binding groove of the neighboring molecule [13, 14, 54]. This ordered assembly is essential for virus replication, as mutations or small molecules that dysregulate oligomerization impair RNP assembly and block virus replication [16, 30–32]. Here we demonstrate that phosphorylation of NP regulates RNP assembly. We identify key residues in NP important for assembly, including a new phosphorylation site, and define the molecular mechanism by which phosphorylation regulates self-association of both influenza virus A and B NP. Mimicking phosphorylation of residues at the NP:NP interface, either at the entrance to the binding groove or in the tail loop, inhibits oligomerization by specifically blocking insertion of the tail loop into the binding groove. Disrupting these residues severely impaired polymerase activity and virus replication. These data support a general model for *Orthomyxoviridae* where the dynamic phosphorylation of NP by host proteins plays a critical role in RNP assembly, and by extension genome replication and successful completion of the virus life cycle (Fig 9).

NP exists in cells as a mixture of monomers and oligomers of varying sizes [55, 56]. Our data confirm that the formation of high-order oligomers occurs spontaneously and does not require other viral proteins or genomic RNA (Fig 5). Mutational analysis performed here identified serine residues that influence the transition of influenza A NP between these two populations (i.e. S165, S407 and S486). Unmodified serines at positions 165 and 407 are required for NP to form higher-order oligomers (Figs 4B and 5). Mutation of these residues to alanine prevents oligomerization, due to the loss of important hydrogen bonds these serines make. By contrast, mutation to alanine of S486, which flanks the entrance to the binding groove, resulted in hyper-oligomerization (Figs 4B and 5). The extreme C-terminus of NP, including S486 and F479 [18], appears to inhibit oligomerization, possibly by reducing binding affinity between protomers or by helping to establish the conformation assumed by the monomeric form [24]. Thus, NP has residues that are required for direct contacts in the homotypic interactions as well as residues that control the assembly process, and single mutations can shift NP to largely monomeric or oligomeric states. Mutants that disturb the equilibrium distribution of NP impair RNP assembly and viral replication (Fig 3C). Thus, both the assembly process and its regulation are critical for successful RNP formation and virus replication.

Stimulation of kinase activity in cells shifted NP towards a monomer, with phosphorylated NP further enriched in the lower molecular weight fractions. Phosphorylation was detected at influenza A NP S165 and S407 and phosphomimetics at these positions all inhibited oligomerization (Figs 2D and 4B). Similarly, a peptide containing S463 in influenza B NP is phosphorylated [41] and phosphomimetics at this position also inhibit binding (Fig 8C) In this scenario, phosphorylation actively blocks protein:protein interactions as phosphorylated residues cannot be accommodated at the interface due to steric clashes. However, phospho-regulation is not absolute as phosphorylated NP was detected in purified RNPs and virions, including phospho-S165 for influenza A NP and phospho-peptides containing S463 for influenza B NP [41]. It is possible the oligomerization is not continuous along the entire length of the RNP [20], allowing phospho-NP to be incorporated at breaks in the NP chain. Additional factors have also been suggested to impact NP oligomerization, including RNA-binding [26], interactions with host proteins [50], secondary NP:NP interaction sites [20, 53] and conformational rearrangements [24]. Thus, phosphorylation is a major regulator of NP oligomerization and may work in tandem with these other processes to tightly control RNP assembly and function.

Our results show that the mechanisms of phospho-regulation are conserved for influenza A and B viruses. We used influenza A and B NP structures to create homology models and structure based alignments for influenza C NP and for NP from the provisionally classified influenza D genera (S5 Fig). These models position phosphorylatable residues in the binding groove and the tail loop at the crucial phosphorylation sites we identified (T169 and S418 for influenza C, and T161 and S416 for influenza D, respectively), as well as the salt-bridge pair at the tail loop-binding grove interface (E354 and R427 for influenza C, and E352 and R425 for influenza D). These interaction sites are well conserved both within and between influenza virus genera, but whether each site is absolutely essential or part of a partially redundant control pathway remains to be determined. It is therefore likely that the regulatory mechanism uncovered here for influenza A and B virus, wherein phosphorylation of NP at the inter-molecular interface blocks oligomerization, is likely shared amongst all influenza virus genera.

The results presented here show that shifting the balance between monomer and oligomer, in either direction, impairs RNP function and reduced the replication of influenza virus. We propose a working model for the regulated assembly of the RNP (Fig 9). A portion of newly synthesized NP is phosphorylated to establish a pool of monomeric, RNA-free NP. Phospho-NP cannot be incorporated into growing RNPs, and might even compete for the polymerase to prevent premature RNP formation. At later times during infection, when genomic RNA synthesis and RNP formation dominates, NP is located to sites of assembly in a non-phosphorylated form. The non-phosphorylated form is then assembled into RNPs aided by the presence of nascent genomic RNA. The reversible nature of phosphorylation establishes a protected pool of monomeric NP that can rapidly transition to become substrate for RNP assembly, consistent with the changing patterns of NP phosphorylation that occur throughout the viral life cycle[37]. Influenza virus encodes neither a kinase nor a phosphatase, therefore it will be important to identify the cellular factors regulating NP phosphorylation as their manipulation might have broad antiviral activity across influenza A, B and C viruses while simultaneously reducing the emergence of resistant viruses by targeting host proteins.

## Materials and Methods

### Plasmids and antibodies

All genes were derived from the influenza A (A/WSN/33) or influenza B (B/Brisbane/60/2008) viruses. pET28a-NΔ7NP was constructed for bacterial expression of protein with a C-terminal His tag and a seven amino acid deletion on the N-terminus, as described [13]. pCDNA3.2-NP-V5 was constructed for eukaryotic expression. Mutations were introduced into the NP gene using the QuickChange mutagenesis kit (Agilent Technologies) and confirmed by sequencing. Polymerase proteins were expressed in cells from the plasmids pCMV-PB2-HA (encoding a C-terminal HA tag), pCDNA3-PA, pCDNA-PA-FLAG and pCDNA3-PB1 [57]. vNA-luc and cNA-luc reporter plasmids encode the firefly luciferase gene flanked by UTRs from the NA gene in the minus or positive sense, respectively [58]. The rescue vectors pTMΔRNP, pBD-PB2, pBD-PB1, pBD-PA and pBD-NP were used to generate recombinant influenza A virus and were based on the influenza reverse genetics system [59, 60]. Recombinant influenza B virus was generated in a similar fashion. Mutations were introduced into pBD-NP by inverse PCR and confirmed by sequencing. GFP:tail loop fusions were generated by inserting coding sequence corresponding to amino acids 402–428 (influenza A NP) or 459–486 (influenza B NP) downstream of GFP in the plasmid pEGFP-C1 (Clontech). Additional sequences were incorporated to encode a cysteine at each end of the tail loop and a four-glycine linker between GFP and the tail loop.

Antibodies used include: anti-HA clone 3F10 (Roche), anti-V5 (R961-25, Invitrogen), anti-GFP (B-2, Santa Cruz Biotech), anti-NP (H16-L10-4R5)[61], anti-FLAG M2 (Sigma) and anti-influenza virus RNP (BEI Resources NR-3133).

### Protein expression, purification, biochemical analysis and electron microscopy

Wild type or mutant NPs were expressed in *E*. *coli* strain Rosetta 2 (DE3) (Novagen) and purified using Ni-NTA affinity (Qiagen). Purified proteins were treated with RNaseA and further purified through a HiTrap *Heparin* HP column (GE Healthcare). Proteins were concentrated to equivalent levels and oligomerization of NP was allowed to reach equilibrium by incubating purified protein at 4°C for 96 hours in buffer containing 50mM Tris, pH7.5, 200mM NaCl and 1mM TCEP. The oligomeric state of NP was subsequently analyzed by size exclusion chromatography through a Superose-6 column calibrated with size standards. For electron microscopy, peak fractions were immediately absorbed on a carbon-coated Cu-grid and stained with a freshly prepared 0.5% Uranyl acetate solution. Images were taken using a Tecnai T12 electron microscope operating at 120 kV with a magnification of 56,000.

To determine the oligomerization state in cells, NP-expressing 293T cells were lysed in 50 mM Tris-HCl, 100 mM KCl, 5 mM MgCl_2_ and 0.5% NP40 containing protease and phosphatase inhibitor cocktails. Where indicated, cells were stimulated with 2.4 μM phorbol 12-myristate 13-acetate (PMA) and 100nM okadaic acid for 2 h before lysis. Total cell extract was clarified by centrifugation, treated with 50 μg/ml of RNaseA for 2 hours at room temperature, and fractionated through a Superose-6 column pre-equilibrated in lysis buffer. Fractions were probed by western blotting.

### Polymerase activity assays and primer extension

293T cells were transfected in triplicate with plasmids encoding PA, PB1, PB2-HA, NP and vNA- or cNA-luciferase reporters. Polymerase activity was measured using the luciferase assay system (Promega) and NP expression was confirmed by western blotting. Primer extensions were performed as described [57].

### Immunoprecipitations

293T cells expressing NP and other interacting partners were lysed in radio-immunoprecipitation assay (RIPA) buffer (50 mM Tris-HCl (pH 7.5), 150 mM NaCl, 2 mM EDTA, 1% NP-40, 0.5% deoxycholate, 0.1% SDS) supplemented with 5mg/ml of BSA and clarified by centrifugation. Lysates were incubated with appropriate antibodies and immunocomplexes were captured on Protein A Dynabeads (Invitrogen). Beads were subsequently washed with RIPA buffer containing 500 mM NaCl and finally in RIPA buffer without BSA. Immunoprecipitates were analyzed by western blotting.

### Rescue of recombinant viruses and multicycle replication assay

Recombinant virus was produced as described by transfecting a co-cultures of 293T and MDBK cells with rescue vectors [62, 63]. Media was replaced 24 hrs later with virus growth media (DMEM, 0.2% bovine serum albumin (BSA), 25 mM HEPES buffer, and 1 μg/ml TPCK trypsin). Virus was subsequently amplified in MDBK or MDCK cells and titered by plaque assay on MDCK cells using a 1.2% Avicel overlay (RC581; FMC Biopolymer)[64]. Multicycle replication assays were performed in triplicate by infection of MDCK cells and viral titers were determined at the indicated time points by plaque assay.

### Mass spectrometry

MDCK cells were infected with WSN (MOI = 5) and samples were collected 2, 4, 6 and 8 hpi. Lysates were prepared in RIPA buffer supplemented with 2 mg/ml BSA and protease and phosphatase inhibitors, pooled, and subject to NP immunoprecipitation as described above. Samples were washed extensively in 10mM Tris pH 7.5, 100mM NaCl and 1mM EDTA and eluted with 8M urea. Purity was confirmed by SDS-PAGE of a small sample of eluted protein and the identity of NP was validated by western blot. The pooled protein sample was reduced with 5 mM dithiothreitol for 30 minutes at 55°C, alkylated with 15 mM iodoacetamide in the dark at ambient temperature for 45 minutes, and quenched by addition of 5 mM dithiothreitol[65]. The protein sample was diluted 1:1 with 50 mM Tris and 5 mM CaCl_2_ and digested with 9 μg tryspin (Promega) overnight at room temperature. Resultant peptides were desalted using a tC18 Sep-Pak cartridge (Waters) and enriched for phosphorylation by immobilized metal affinity chromatography (IMAC) using Ni-NTA magnetic agarose beads (Qiagen)[66]. Both non-phosphorylated and phosphorylated peptide samples were resuspended in 14 μL of 0.2% formic acid and analyzed by mass spectrometry (MS). An 80 minute nano-liquid chromatography (nLC) gradient was used to introduce peptides to an Oribtrap Elite mass spectrometer (Thermo Scientific). Preliminary MS experiments used data dependent acquisition (DDA) to discover IMAC-enriched peptides which were present in the sample, using either collisonally activated dissociation (CAD) or higher-energy collisonial dissociation (HCD) to fragment eluting peptides[67]. Spectra obtained from these DDA experiments were searched against a concatenated target-decoy database containing the protein sequences of Canis familiaris and Influenza A (Uniprot) using Sequest within the Proteome Discoverer software package (Thermo Fisher). For all samples, cysteine carbamidomethylation and methionine oxidation were searched as fixed and variable amino acid modifications, respectively, and phosphorylation of serine, threonine, and tyrosine residues were searched as variable modifications. Precursor mass tolerance was defined as 40 ppm and fragment ion tolerance was set to 0.30 Da (ion trap MS/MS) and 0.02 Da (FT MS/MS)[68]. Search results were filtered to 1% false discovery rate (FDR) using precursor mass error. PhosphoRS[69] was used to localize phosphorylation to amino acid residues using a fragment mass tolerance of 0.02 Da, automatically considering neutral loss peaks for HCD and considering a maximum of 200 maximum position isoforms per phosphopeptide. Using the untargeted DDA MS approach, a singly phosphorylated peptide corresponding to site S165 was identified from the IMAC enriched sample, mapping to the sequence 163-MCpSLMQGSTLPR-174. Additionally, several peptide-spectral matches mapping to a singly phosphorylated peptide, 401-ASSGQISIQPTFSVQR-416, were identified from the untargeted MS experiments. Follow-up, targeted MS runs were used to isolate only the ASSGQISIQPTFSVQR peptide *m/z* values corresponding to the phosphorylated peptide. Using targeted CAD or HCD, four distinct phosphoisoforms of the peptide were observed, with phosphorylations localized to the S402, S403, S407, and S413 residues.

### Statistics

Data are presented as the mean +/- standard deviation (n≥3). For polymerase activity assays, data were normalized to WT and error was propagated throughout to yield normalized standard deviation.

## Supporting Information

S1 Table Theoretical and observed sequencing ions, phosphorylation losses and neutral losses from the peptide MCpSLMQGSTLPR (pS165). Theoretical fragments are in black, whereas observed b fragments are highlighted in red and observed y fragments are in blue.
(TIF)

S2 Table Theoretical and observed sequencing ions, phosphorylation losses and neutral losses from the peptide ApSSGQISIQPTFSVQR (pS402). Theoretical fragments are in black, whereas observed b fragments are highlighted in red and observed y fragments are in blue.
(TIF)

S3 Table Theoretical and observed sequencing ions, phosphorylation losses and neutral losses from the peptide ASpSGQISIQPTFSVQR (pS403). Theoretical fragments are in black, whereas observed b fragments are highlighted in red and observed y fragments are in blue.
(TIF)

S4 Table Theoretical and observed sequencing ions, phosphorylation losses and neutral losses from the peptide ASSGQIpSIQPTFSVQR (pS407). Theoretical fragments are in black, whereas observed b fragments are highlighted in red and observed y fragments are in blue.
(TIF)

S5 Table Theoretical and observed sequencing ions, phosphorylation losses and neutral losses from the peptide ASSGQISIQPTFpSVQR (pS413). Theoretical fragments are in black, whereas observed b fragments are highlighted in red and observed y fragments are in blue.
(TIF)

S1 Fig Dynamic subcellular localization of WT and mutant NP. HeLa cells were transfected with plasmids encoding wild-type or mutant NP-V5, fixed at 10 or 16 h post-transfection, and visualized by immunofluorescence using anti-V5 antibody.
(TIF)

S2 Fig NP phosphorylation sites by identified by mass spectrometry. (a) purified protein subject to mass spectrometry. (b) Targeted mass spectrometry was used to localize phosphorylation to singly-phosphorylated peptides which were enriched from a sample of purified NP protein. Three distinct phosphoisoforms of the peptide ASSGQISIQPTFSVQR were identified from the purified NP sample in addition to the Ser407 phosphopeptide. Phosphorylation was localized to serines (B) S402, and (C) S403 from influenza NP. (D) An additional mono-phosphorylated peptide was observed localizing to Ser165, a confirmation of previous studies.
(TIF)

S3 Fig Quantification of primer extension analysis presented in Fig 3B. Intensity of vRNA, mRNA and cRNA products, denoted by rectangular boxes, were quantified for phospho-mutants (A) and phosphomimetic NP using ImageJ. Band intensities were plotted as a relative percentage of WT NP.
(TIF)

S4 Fig Electron micrographs of oligomeric WT NP and NP 486A. Oligomeric NP was isolated by size exclusion chromatography and immediately prepared for electron microscopy by negative staining with uranyl acetate. Images were taken at 56,000x magnification. Images shown here are the source of the isolated oligomers shown in Fig 4.
(TIF)

S5 Fig Phosphorylated serine residues in influenza virus A NP are conserved in in all influenza virus genera. Structures of influenza virus A (A/WSN/1933/H1N1, PDB:2IQH) and B (B/Managua/4577.01/2008, PDB:3TJ0) NP were used as templates for homology modeling of influenza C (C/Ann Arbor/50) and D (D/swine/Oklahoma/1334/2011) using Phyre2. The resultant structure-based alignment is shown. Conserved phosphorylation sites S165 and S407 in influenza A NP align with S226 and S463 in B NP, T169 and S418 in C NP, and T161 and S416 for D NP. Conserved secondary structure helices (h) and beta strands (e) are indicated. Alignment was created with PROMALS3D. Note that sequences derived from these structures do not contain complete N- and C-termini.
(TIF)

## References

[1] Taubenberger JK, Morens DM. The pathology of influenza virus infections. Annual review of pathology. 2008;3:499–522. 10.1146/annurev.pathmechdis.3.121806.154316 18039138 PMC2504709

[2] Palese P, Shaw M. Orthomyxoviridae: The Viruses and Their Replication. Fields Virology, Volume 2 (eds Knipe DM and Howley PM). 2001:1647–89.

[3] Mehle A, McCullers JA. Structure and function of the influenza virus replication machinery and PB1-F2 In: Webster RG, Monto AS, Braciale TJ, Lamb RA, editors. Textbook of Influenza, 2nd Edition Oxford, UK: John Wiley & Sons, Ltd; 2013 p. 133–45.

[4] Hutchinson EC, Fodor E. Transport of the influenza virus genome from nucleus to nucleus. Viruses. 2013;5(10):2424–46. 10.3390/v5102424 24104053 PMC3814596

[5] Plotch SJ, Bouloy M, Ulmanen I, Krug RM. A unique cap(m7GpppXm)-dependent influenza virion endonuclease cleaves capped RNAs to generate the primers that initiate viral RNA transcription. Cell. 1981;23(3):847–58. 6261960 10.1016/0092-8674(81)90449-9

[6] Jorba N, Coloma R, Ortin J. Genetic trans-complementation establishes a new model for influenza virus RNA transcription and replication. PLoS Pathog. 2009;5(5):e1000462 10.1371/journal.ppat.1000462 19478885 PMC2682650

[7] Vreede FT, Jung TE, Brownlee GG. Model suggesting that replication of influenza virus is regulated by stabilization of replicative intermediates. J Virol. 2004;78(17):9568–72. 10.1128/jvi.78.17.9568–9572.2004 15308750 PMC506943

[8] Moeller A, Kirchdoerfer RN, Potter CS, Carragher B, Wilson IA. Organization of the influenza virus replication machinery. Science. 2012;338(6114):1631–4. 10.1126/science.1227270 23180774 PMC3578580

[9] Arranz R, Coloma R, Chichon FJ, Conesa JJ, Carrascosa JL, Valpuesta JM, et al The structure of native influenza virion ribonucleoproteins. Science. 2012;338(6114):1634–7. 10.1126/science.1228172 23180776

[10] Pons MW, Schulze IT, Hirst GK. Isolation and characterization of the ribonucleoprotein of influenza virus. Virology. 1969;39(2):250–9. 10.1016/0042-6822(69)90045-2 4186524

[11] Klumpp K, Ruigrok RW, Baudin F. Roles of the influenza virus polymerase and nucleoprotein in forming a functional RNP structure. Embo J. 1997;16(6):1248–57. 9135141 10.1093/emboj/16.6.1248 PMC1169723

[12] York A, Hengrung N, Vreede FT, Huiskonen JT, Fodor E. Isolation and characterization of the positive-sense replicative intermediate of a negative-strand RNA virus. Proc Natl Acad Sci U S A. 2013;110(45):E4238–45. 10.1073/pnas.1315068110 24145413 PMC3831450

[13] Ye Q, Krug RM, Tao YJ. The mechanism by which influenza A virus nucleoprotein forms oligomers and binds RNA. Nature. 2006;444(7122):1078–82. 10.1038/nature05379 17151603

[14] Ng AK, Zhang H, Tan K, Li Z, Liu JH, Chan PK, et al Structure of the influenza virus A H5N1 nucleoprotein: implications for RNA binding, oligomerization, and vaccine design. Faseb J. 2008;22(10):3638–47. 10.1096/fj.08-112110 18614582 PMC2537428

[15] Chan WH, Ng AK, Robb NC, Lam MK, Chan PK, Au SW, et al Functional analysis of the influenza virus H5N1 nucleoprotein tail loop reveals amino acids that are crucial for oligomerization and ribonucleoprotein activities. J Virol. 2010;84(14):7337–45. 10.1128/JVI.02474-09 20463064 PMC2898228

[16] Shen Y-F, Chen Y-H, Chu S-Y, Lin M-I, Hsu H-T, Wu P-Y, et al E339…R416 salt bridge of nucleoprotein as a feasible target for influenza virus inhibitors. Proceedings of the National Academy of Sciences. 2011;108(40):16515–20. 10.1073/pnas.1113107108 21930946 PMC3189076

[17] Coloma R, Valpuesta JM, Arranz R, Carrascosa JL, Ortin J, Martin-Benito J. The structure of a biologically active influenza virus ribonucleoprotein complex. PLoS Pathog. 2009;5(6):e1000491 10.1371/journal.ppat.1000491 19557158 PMC2695768

[18] Elton D, Medcalf E, Bishop K, Digard P. Oligomerization of the influenza virus nucleoprotein: identification of positive and negative sequence elements. Virology. 1999;260(1):190–200. 10.1006/viro.1999.9818 10405371

[19] Marklund JK, Ye Q, Dong J, Tao YJ, Krug RM. Sequence in the Influenza A Virus Nucleoprotein Required for Viral Polymerase Binding and RNA Synthesis. Journal of Virology. 2012;86(13):7292–7. 10.1128/jvi.00014-12 22532672 PMC3416340

[20] Ye Q, Guu TSY, Mata DA, Kuo R-L, Smith B, Krug RM, et al Biochemical and Structural Evidence in Support of a Coherent Model for the Formation of the Double-Helical Influenza A Virus Ribonucleoprotein. MBio. 2013;4(1). 10.1128/mBio.00467-12 PMC3531806 23269829

[21] Resa-Infante P, Jorba N, Coloma R, Ortin J. The influenza virus RNA synthesis machine: advances in its structure and function. RNA Biol. 2011;8(2):207–15. 21358279 10.4161/rna.8.2.14513 PMC3127100

[22] Tao YJ, Ye Q. RNA virus replication complexes. PLoS Pathog. 2010;6(7):e1000943 10.1371/journal.ppat.1000943 20661480 PMC2908549

[23] Zheng W, Tao YJ. Structure and assembly of the influenza A virus ribonucleoprotein complex. FEBS Lett. 2013;587(8):1206–14. 10.1016/j.febslet.2013.02.048 23499938

[24] Chenavas S, Estrozi LF, Slama-Schwok A, Delmas B, Di Primo C, Baudin F, et al Monomeric nucleoprotein of influenza A virus. PLoS Pathog. 2013;9(3):e1003275 10.1371/journal.ppat.1003275 23555270 PMC3610751

[25] Vreede FT, Ng AK, Shaw PC, Fodor E. Stabilization of influenza virus replication intermediates is dependent on the RNA-binding but not the homo-oligomerization activity of the viral nucleoprotein. J Virol. 2011;85(22):12073–8. 10.1128/jvi.00695-11 21917965 PMC3209277

[26] Yamanaka K, Ishihama A, Nagata K. Reconstitution of influenza virus RNA-nucleoprotein complexes structurally resembling native viral ribonucleoprotein cores. The Journal of biological chemistry. 1990;265(19):11151–5. 2358455

[27] Turrell L, Lyall JW, Tiley LS, Fodor E, Vreede FT. The role and assembly mechanism of nucleoprotein in influenza A virus ribonucleoprotein complexes. Nature communications. 2013;4:1591 10.1038/ncomms2589 23481399 PMC4168216

[28] Baudin F, Bach C, Cusack S, Ruigrok RW. Structure of influenza virus RNP. I. Influenza virus nucleoprotein melts secondary structure in panhandle RNA and exposes the bases to the solvent. Embo J. 1994;13(13):3158–65. 8039508 10.1002/j.1460-2075.1994.tb06614.x PMC395207

[29] Tarus B, Bakowiez O, Chenavas S, Duchemin L, Estrozi LF, Bourdieu C, et al Oligomerization paths of the nucleoprotein of influenza A virus. Biochimie. 2012;94(3):776–85. 10.1016/j.biochi.2011.11.009 22155087

[30] Su CY, Cheng TJ, Lin MI, Wang SY, Huang WI, Lin-Chu SY, et al High-throughput identification of compounds targeting influenza RNA-dependent RNA polymerase activity. Proc Natl Acad Sci U S A. 2010;107(45):19151–6. 10.1073/pnas.1013592107 20974907 PMC2984200

[31] Gerritz SW, Cianci C, Kim S, Pearce BC, Deminie C, Discotto L, et al Inhibition of influenza virus replication via small molecules that induce the formation of higher-order nucleoprotein oligomers. Proc Natl Acad Sci U S A. 2011;108(37):15366–71. 10.1073/pnas.1107906108 21896751 PMC3174639

[32] Kao RY, Yang D, Lau L-S, Tsui WHW, Hu L, Dai J, et al Identification of influenza A nucleoprotein as an antiviral target. Nat Biotech. 2010;28(6):600–5. 10.1038/nbt.1638 20512121 PMC7097325

[33] Masters PS, Banerjee AK. Complex formation with vesicular stomatitis virus phosphoprotein NS prevents binding of nucleocapsid protein N to nonspecific RNA. J Virol. 1988;62(8):2658–64. 2839693 10.1128/jvi.62.8.2658-2664.1988 PMC253697

[34] Curran J, Marq JB, Kolakofsky D. An N-terminal domain of the Sendai paramyxovirus P protein acts as a chaperone for the NP protein during the nascent chain assembly step of genome replication. J Virol. 1995;69(2):849–55. 7815552 10.1128/jvi.69.2.849-855.1995 PMC188651

[35] Mavrakis M, Méhouas S, Réal E, Iseni F, Blondel D, Tordo N, et al Rabies virus chaperone: identification of the phosphoprotein peptide that keeps nucleoprotein soluble and free from non-specific RNA. Virology. 2006;349(2):422–9. 10.1016/j.virol.2006.01.030 16494915

[36] Mondal A, Roy A, Sarkar S, Mukherjee J, Ganguly T, Chattopadhyay D. Interaction of chandipura virus N and P proteins: identification of two mutually exclusive domains of N involved in interaction with P. PLoS One. 2012;7(4):e34623 10.1371/journal.pone.0034623 22485180 PMC3317646

[37] Kistner O, Muller K, Scholtissek C. Differential phosphorylation of the nucleoprotein of influenza A viruses. J Gen Virol. 1989;70 (Pt 9):2421–31. 2778438 10.1099/0022-1317-70-9-2421

[38] Kistner O, Muller H, Becht H, Scholtissek C. Phosphopeptide fingerprints of nucleoproteins of various influenza A virus strains grown in different host cells. J Gen Virol. 1985;66 (Pt 3):465–72. 3882883 10.1099/0022-1317-66-3-465

[39] Privalsky ML, Penhoet EE. Phosphorylated protein component present in influenza virions. J Virol. 1977;24(1):401–5. 904030 10.1128/jvi.24.1.401-405.1977 PMC515941

[40] Privalsky ML, Penhoet EE. The structure and synthesis of influenza virus phosphoproteins. The Journal of biological chemistry. 1981;256(11):5368–76. 7240143

[41] Hutchinson EC, Denham EM, Thomas B, Trudgian DC, Hester SS, Ridlova G, et al Mapping the phosphoproteome of influenza A and B viruses by mass spectrometry. PLoS Pathog. 2012;8(11):e1002993 10.1371/journal.ppat.1002993 23144613 PMC3493474

[42] Liao TL, Wu CY, Su WC, Jeng KS, Lai MM. Ubiquitination and deubiquitination of NP protein regulates influenza A virus RNA replication. Embo J. 2010;29(22):3879–90. 10.1038/emboj.2010.250 20924359 PMC2989104

[43] Arrese M, Portela A. Serine 3 is critical for phosphorylation at the N-terminal end of the nucleoprotein of influenza virus A/Victoria/3/75. J Virol. 1996;70(6):3385–91. 8648669 10.1128/jvi.70.6.3385-3391.1996 PMC190210

[44] Sasaki Y, Hagiwara K, Kakisaka M, Yamada K, Murakami T, Aida Y. Importin α3/Qip1 is involved in multiplication of mutant influenza virus with alanine mutation at amino acid 9 independently of nuclear transport function. PLoS One. 2013;8(1):e55765 10.1371/journal.pone.0055765 23383277 PMC3559588

[45] Mena I, Jambrina E, Albo C, Perales B, Ortin J, Arrese M, et al Mutational analysis of influenza A virus nucleoprotein: identification of mutations that affect RNA replication. J Virol. 1999;73(2):1186–94. 9882320 10.1128/jvi.73.2.1186-1194.1999 PMC103939

[46] Li Z, Watanabe T, Hatta M, Watanabe S, Nanbo A, Ozawa M, et al Mutational analysis of conserved amino acids in the influenza A virus nucleoprotein. J Virol. 2009;83(9):4153–62. 10.1128/jvi.02642-08 19225007 PMC2668439

[47] Turrell L, Hutchinson EC, Vreede FT, Fodor E. Regulation of influenza a virus nucleoprotein oligomerization by phosphorylation. J Virol. 2015;89(2):1452–5. 10.1128/jvi.02332-14 25355893 PMC4300657

[48] Chenavas S, Crepin T, Delmas B, Ruigrok RW, Slama-Schwok A. Influenza virus nucleoprotein: structure, RNA binding, oligomerization and antiviral drug target. Future microbiology. 2013;8:1537–45. 10.2217/fmb.13.128 24266354

[49] Momose F, Basler CF, O'Neill RE, Iwamatsu A, Palese P, Nagata K. Cellular splicing factor RAF-2p48/NPI-5/BAT1/UAP56 interacts with the influenza virus nucleoprotein and enhances viral RNA synthesis. J Virol. 2001;75(4):1899–908. 11160689 10.1128/JVI.75.4.1899-1908.2001 PMC115136

[50] Boulo S, Akarsu H, Lotteau V, Muller CW, Ruigrok RW, Baudin F. Human importin alpha and RNA do not compete for binding to influenza A virus nucleoprotein. Virology. 2011;409(1):84–90. 10.1016/j.virol.2010.10.001 20974480

[51] Sharma S, Mayank AK, Nailwal H, Tripathi S, Patel JR, Bowzard JB, et al Influenza A viral nucleoprotein interacts with cytoskeleton scaffolding protein α-actinin-4 for viral replication. FEBS J. 2014;281(13):2899–914. 10.1111/febs.12828 24802111 PMC7164065

[52] Biswas SK, Boutz PL, Nayak DP. Influenza virus nucleoprotein interacts with influenza virus polymerase proteins. J Virol. 1998;72(7):5493–501. 9621005 10.1128/jvi.72.7.5493-5501.1998 PMC110190

[53] Ng AK, Lam MK, Zhang H, Liu J, Au SW, Chan PK, et al Structural basis for RNA binding and homo-oligomer formation by influenza B virus nucleoprotein. J Virol. 2012;86(12):6758–67. 10.1128/JVI.00073-12 22496219 PMC3393550

[54] Zheng W, Olson J, Vakharia V, Tao YJ. The crystal structure and RNA-binding of an orthomyxovirus nucleoprotein. PLoS Pathog. 2013;9(9):e1003624 10.1371/journal.ppat.1003624 24068932 PMC3771910

[55] Prokudina-Kantorovich EN, Semenova NP. Intracellular Oligomerization of Influenza Virus Nucleoprotein. Virology. 1996;223(1):51–6. 10.1006/viro.1996.0454. 8806539

[56] Zhao H, Ekstrom M, Garoff H. The M1 and NP proteins of influenza A virus form homo- but not heterooligomeric complexes when coexpressed in BHK-21 cells. Journal of General Virology. 1998;79(10):2435–46. 9780049 10.1099/0022-1317-79-10-2435

[57] Mehle A, Doudna JA. An inhibitory activity in human cells restricts the function of an avian-like influenza virus polymerase. Cell Host Microbe. 2008;4(2):111–22. 10.1016/j.chom.2008.06.007 18692771 PMC2597520

[58] Regan JF, Liang Y, Parslow TG. Defective assembly of influenza A virus due to a mutation in the polymerase subunit PA. J Virol. 2006;80(1):252–61. 10.1128/JVI.80.1.252–261.2006 16352550 PMC1317532

[59] Hoffmann E, Neumann G, Kawaoka Y, Hobom G, Webster RG. A DNA transfection system for generation of influenza A virus from eight plasmids. Proc Natl Acad Sci U S A. 2000;97(11):6108–13. 10.1073/pnas.100133697 10801978 PMC18566

[60] Neumann G, Fujii K, Kino Y, Kawaoka Y. An improved reverse genetics system for influenza A virus generation and its implications for vaccine production. Proc Natl Acad Sci U S A. 2005;102(46):16825–9. 10.1073/pnas.0505587102 16267134 PMC1283806

[61] Yewdell JW, Frank E, Gerhard W. Expression of influenza A virus internal antigens on the surface of infected P815 cells. J Immunol. 1981;126(5):1814–9. 7217668

[62] Kirui J, Bucci MD, Poole DS, Mehle A. Conserved features of the PB2 627 domain impact influenza virus polymerase function and replication. J Virol. 2014;88(11):5977–86. 10.1128/JVI.00508-14 24623411 PMC4093881

[63] Tran V, Moser LA, Poole DS, Mehle A. Highly sensitive real-time in vivo imaging of an influenza reporter virus reveals dynamics of replication and spread. J Virol. 2013;87(24):13321–9. 10.1128/JVI.02381-13 24089552 PMC3838222

[64] Matrosovich M, Matrosovich T, Garten W, Klenk HD. New low-viscosity overlay medium for viral plaque assays. Virology journal. 2006;3:63 10.1186/1743-422X-3-63 16945126 PMC1564390

[65] Richards AL, Vincent CE, Guthals A, Rose CM, Westphall MS, Bandeira N, et al Neutron-encoded signatures enable product ion annotation from tandem mass spectra. Mol Cell Proteomics. 2013;12(12):3812–23. 10.1074/mcp.M113.028951 24043425 PMC3861726

[66] Rose CM, Venkateshwaran M, Volkening JD, Grimsrud PA, Maeda J, Bailey DJ, et al Rapid phosphoproteomic and transcriptomic changes in the rhizobia-legume symbiosis. Mol Cell Proteomics. 2012;11(9):724–44. 10.1074/mcp.M112.019208 22683509 PMC3434772

[67] Vincent CE, Potts GK, Ulbrich A, Westphall MS, Atwood JA, Coon JJ, et al Segmentation of Precursor Mass Range Using "Tiling" Approach Increases Peptide Identifications for MS1-Based Label-Free Quantification. Anal Chem. 2013;85(5):2825–32. 10.1021/Ac303352n 23350991 PMC3607285

[68] Hebert AS, Richards AL, Bailey DJ, Ulbrich A, Coughlin EE, Westphall MS, et al The one hour yeast proteome. Mol Cell Proteomics. 2014;13(1):339–47. 10.1074/mcp.M113.034769 24143002 PMC3879625

[69] Taus T, Kocher T, Pichler P, Paschke C, Schmidt A, Henrich C, et al Universal and confident phosphorylation site localization using phosphoRS. J Proteome Res. 2011;10(12):5354–62. 10.1021/pr200611n 22073976

