# Supplementary figures and images for: Phosphorylation at the Homotypic Interface Regulates Nucleoprotein Oligomerization and Assembly of the Influenza Virus Replication Machinery
Source: PLoS Pathog. 2015 Apr 13;11(4):e1004826. doi: 10.1371/journal.ppat.1004826 (PMC4395114; doi:10.1371/journal.ppat.1004826)

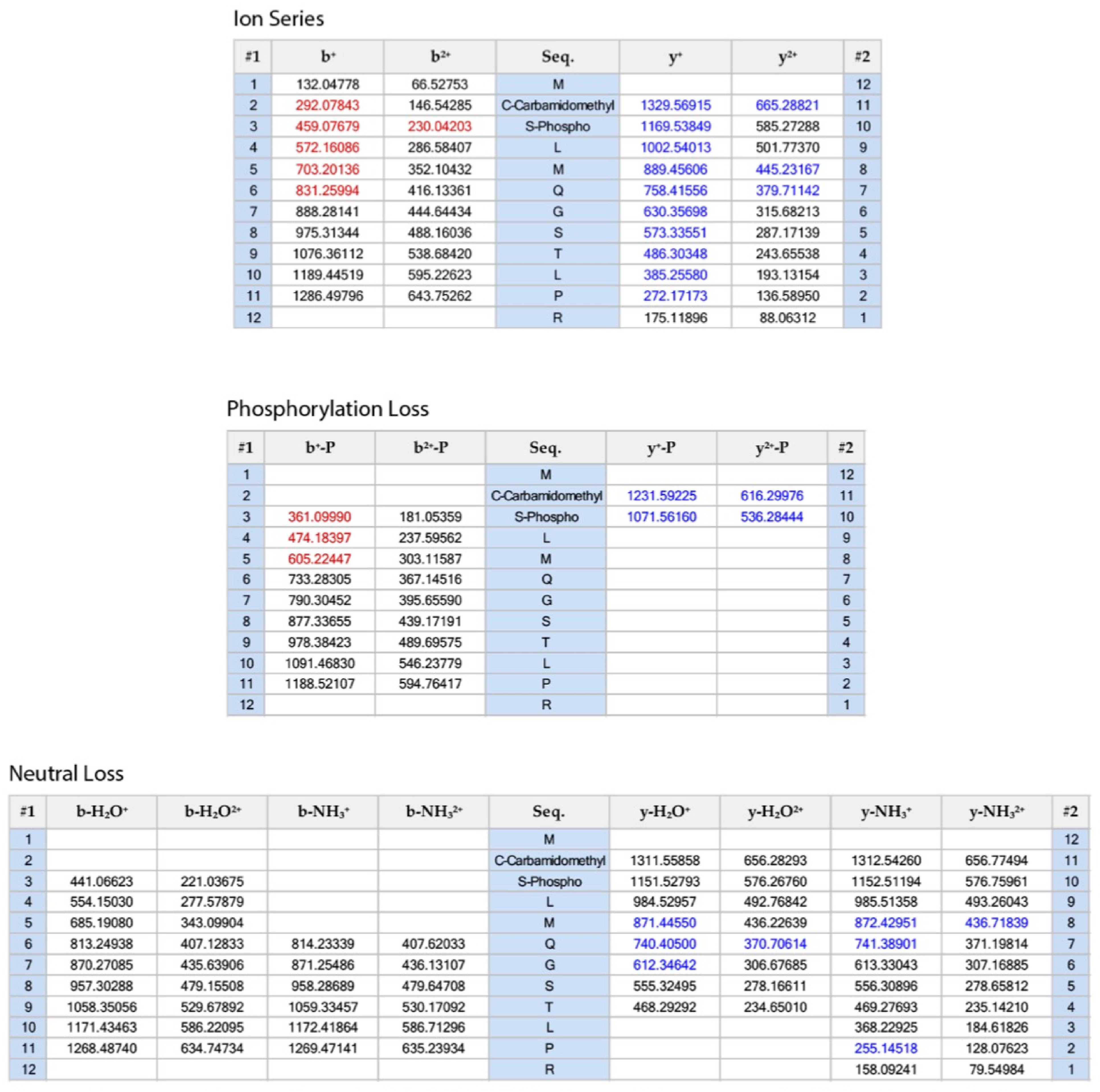

Supplement: S1 Table — Theoretical fragments are in black, whereas observed b fragments are highlighted in red and observed y fragments are in blue. (TIF) [file ppat.1004826.s001.tif]

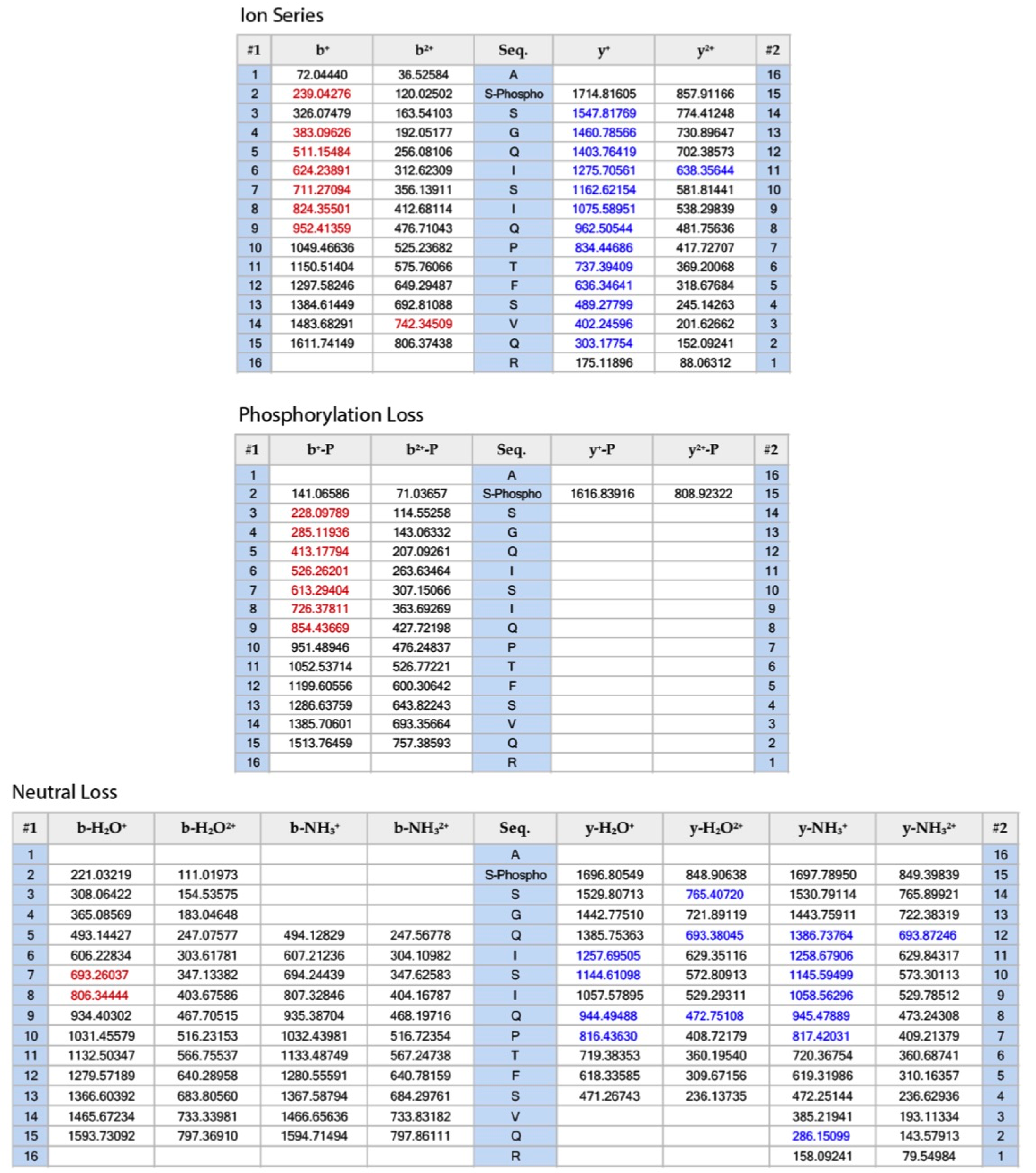

Supplement: S2 Table — Theoretical fragments are in black, whereas observed b fragments are highlighted in red and observed y fragments are in blue. (TIF) [file ppat.1004826.s002.tif]

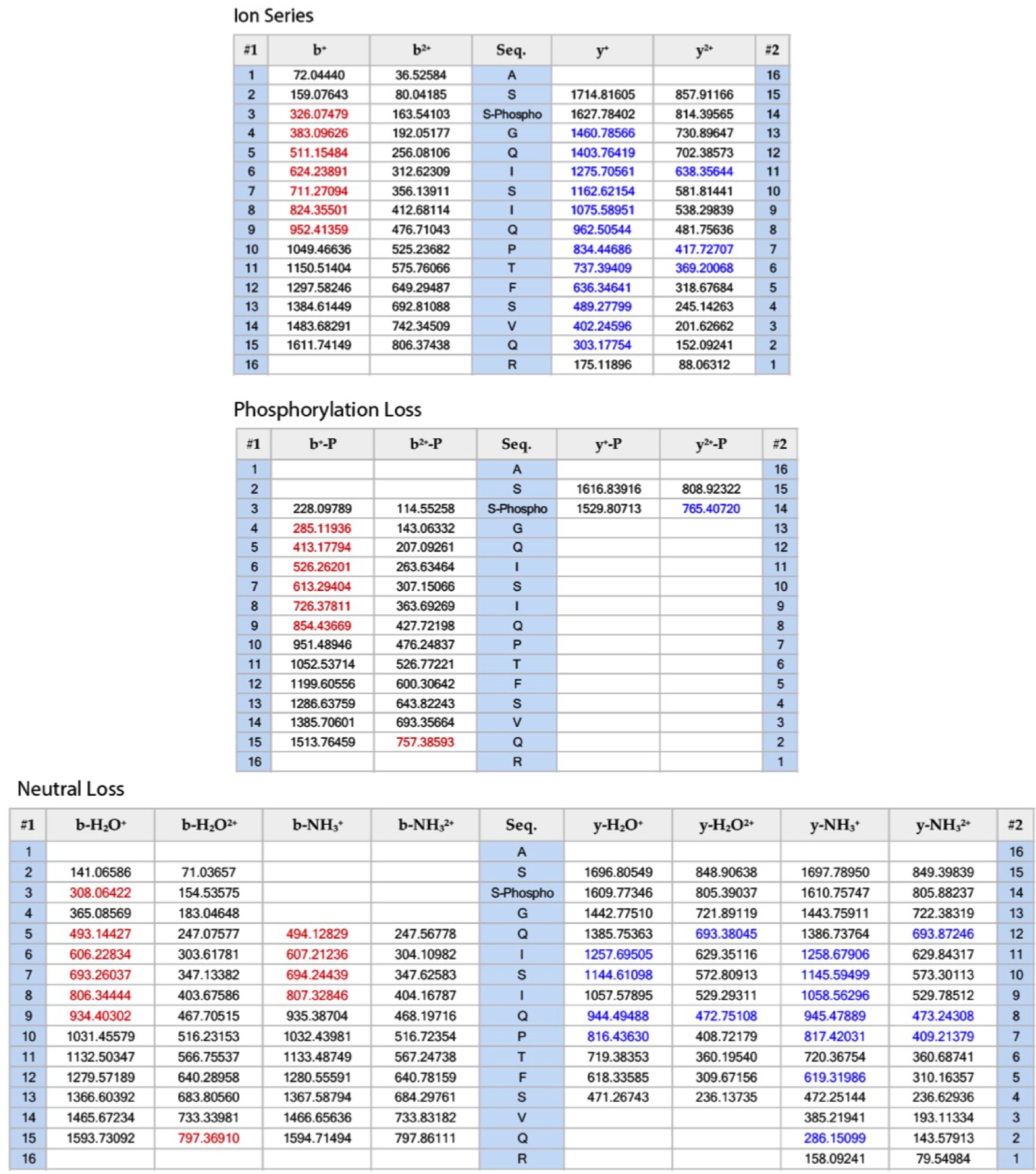

Supplement: S3 Table — Theoretical fragments are in black, whereas observed b fragments are highlighted in red and observed y fragments are in blue. (TIF) [file ppat.1004826.s003.tif]

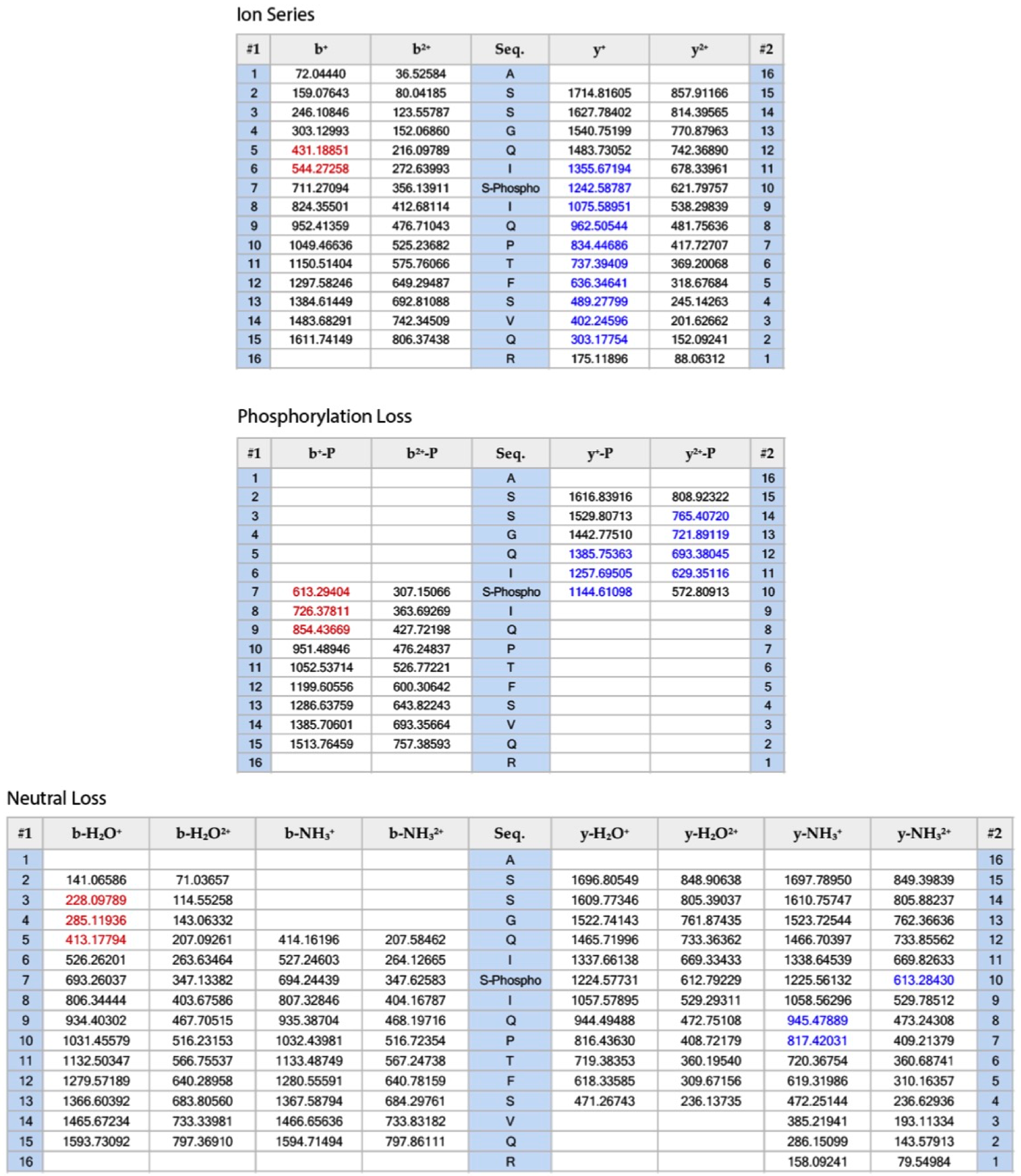

Supplement: S4 Table — Theoretical fragments are in black, whereas observed b fragments are highlighted in red and observed y fragments are in blue. (TIF) [file ppat.1004826.s004.tif]

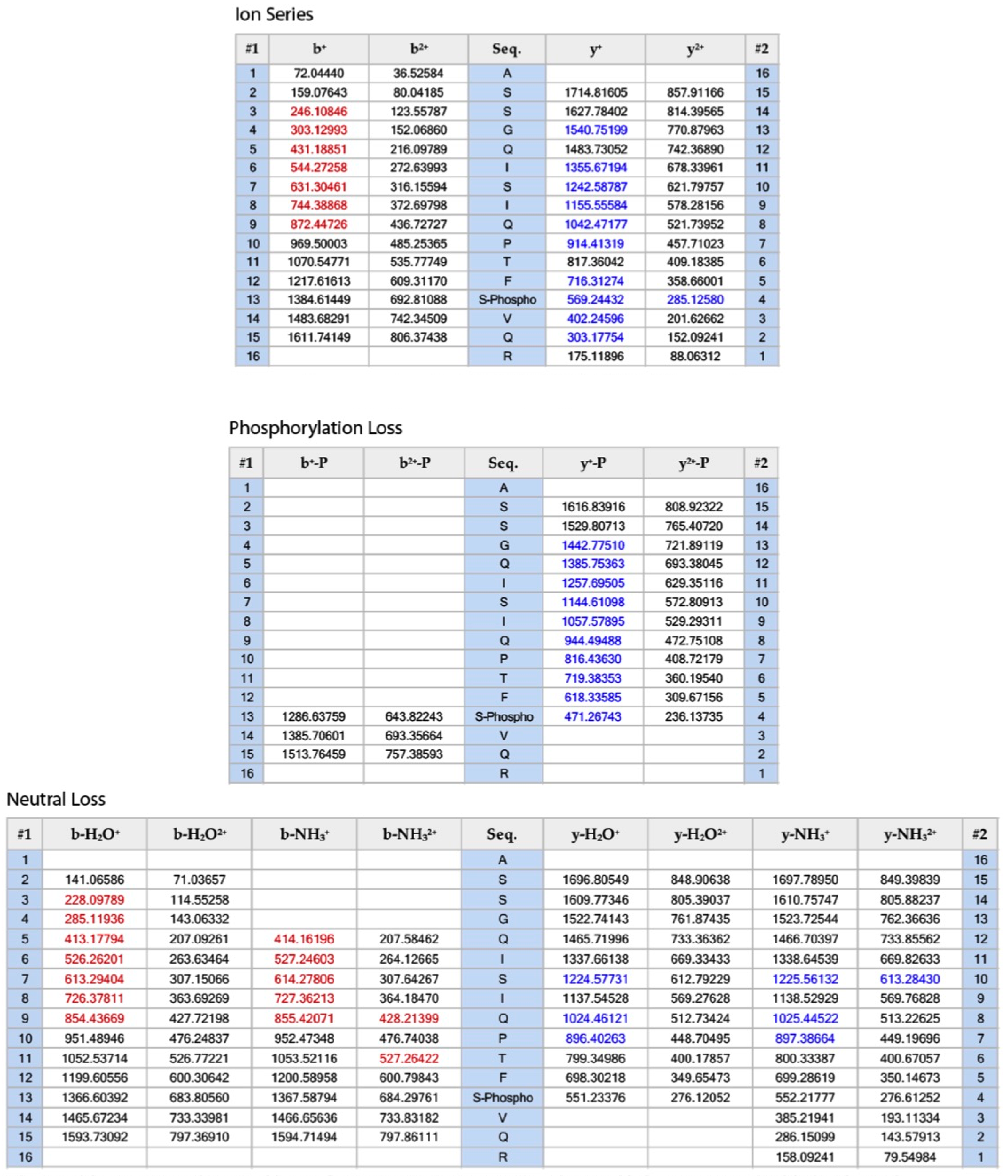

Supplement: S5 Table — Theoretical fragments are in black, whereas observed b fragments are highlighted in red and observed y fragments are in blue. (TIF) [file ppat.1004826.s005.tif]

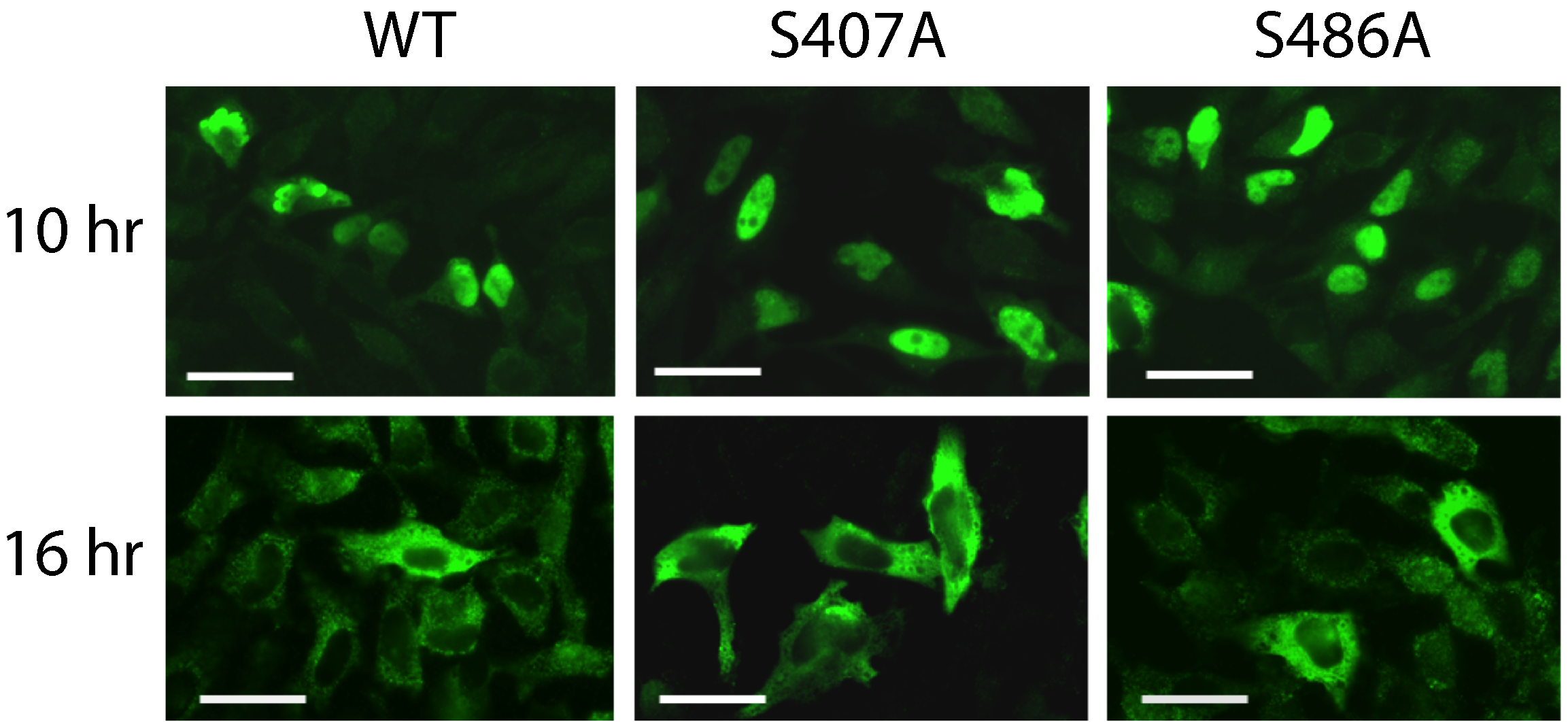

Supplement: S1 Fig — HeLa cells were transfected with plasmids encoding wild-type or mutant NP-V5, fixed at 10 or 16 h post-transfection, and visualized by immunofluorescence using anti-V5 antibody. (TIF) [file ppat.1004826.s006.tif]

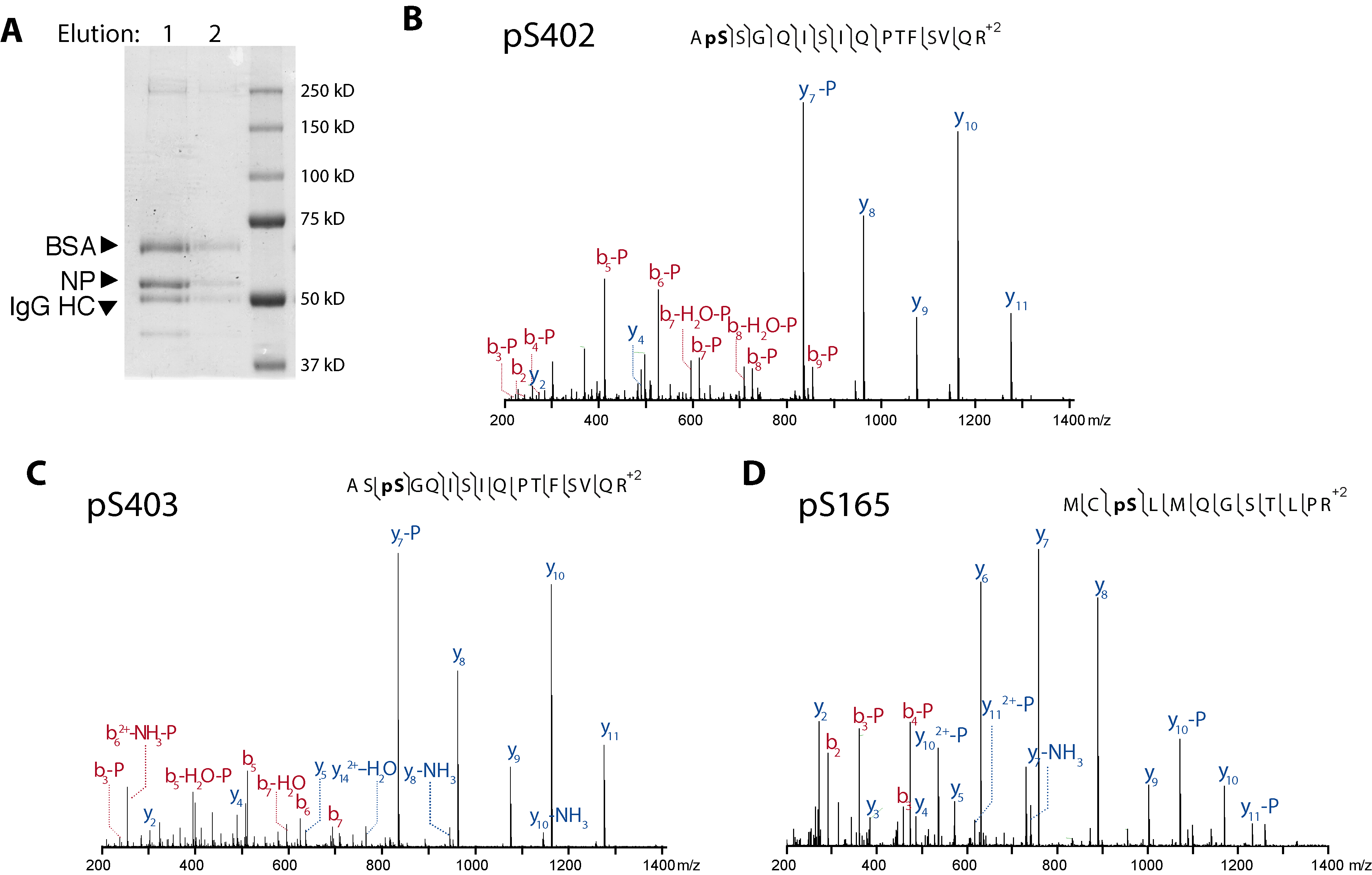

Supplement: S2 Fig — (a) purified protein subject to mass spectrometry. (b) Targeted mass spectrometry was used to localize phosphorylation to singly-phosphorylated peptides which were enriched from a sample of purified NP protein. Three distinct phosphoisoforms of the peptide ASSGQISIQPTFSVQR were identified from the purified NP sample in addition to the Ser407 phosphopeptide. Phosphorylation was localized to serines (B) S402, and (C) S403 from influenza NP. (D) An additional mono-phosphorylated peptide was observed localizing to Ser165, a confirmation of previous studies. (TIF) [file ppat.1004826.s007.tif]

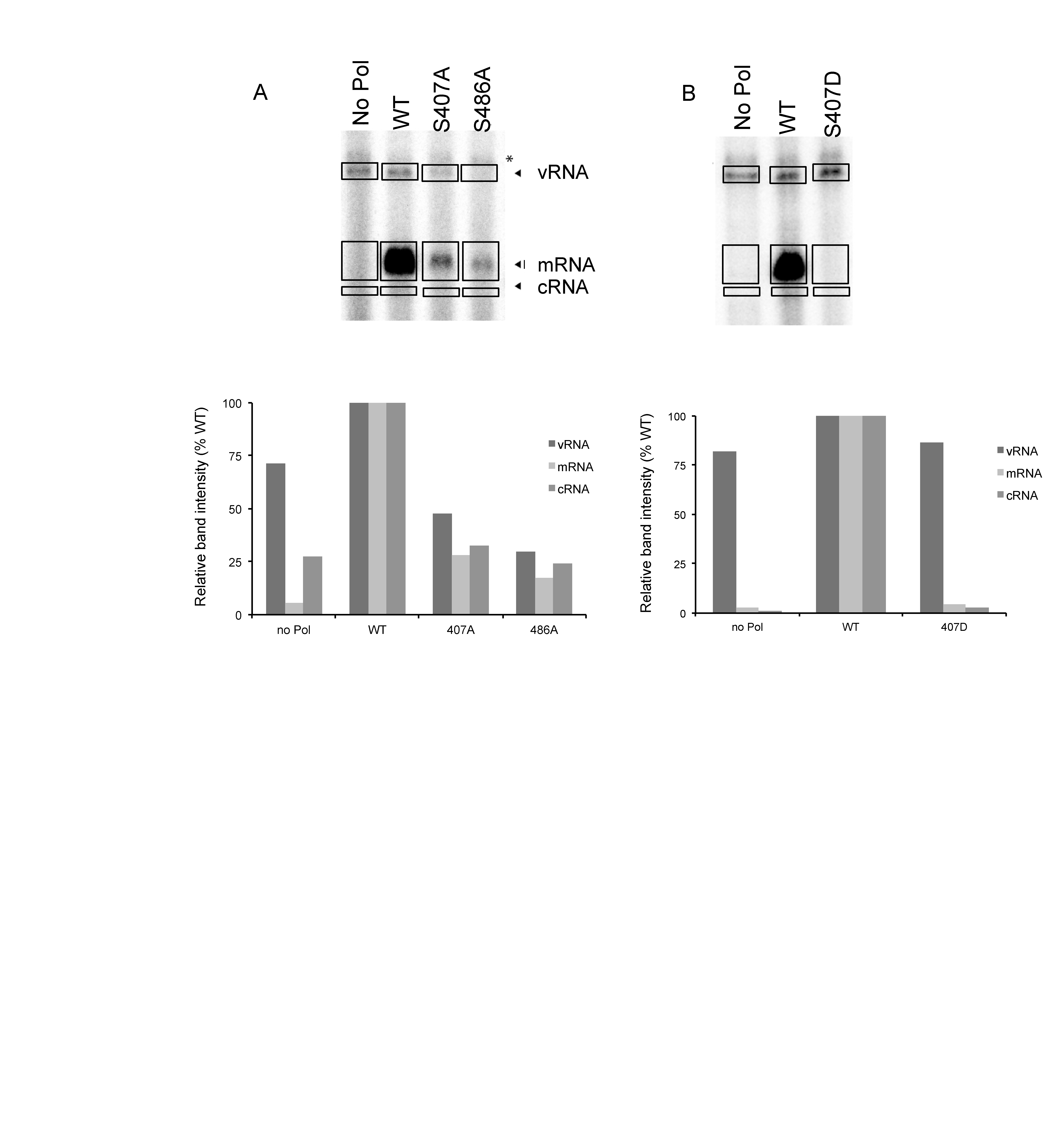

Supplement: S3 Fig — Intensity of vRNA, mRNA and cRNA products, denoted by rectangular boxes, were quantified for phospho-mutants (A) and phosphomimetic NP using ImageJ. Band intensities were plotted as a relative percentage of WT NP. (TIF) [file ppat.1004826.s008.tif]

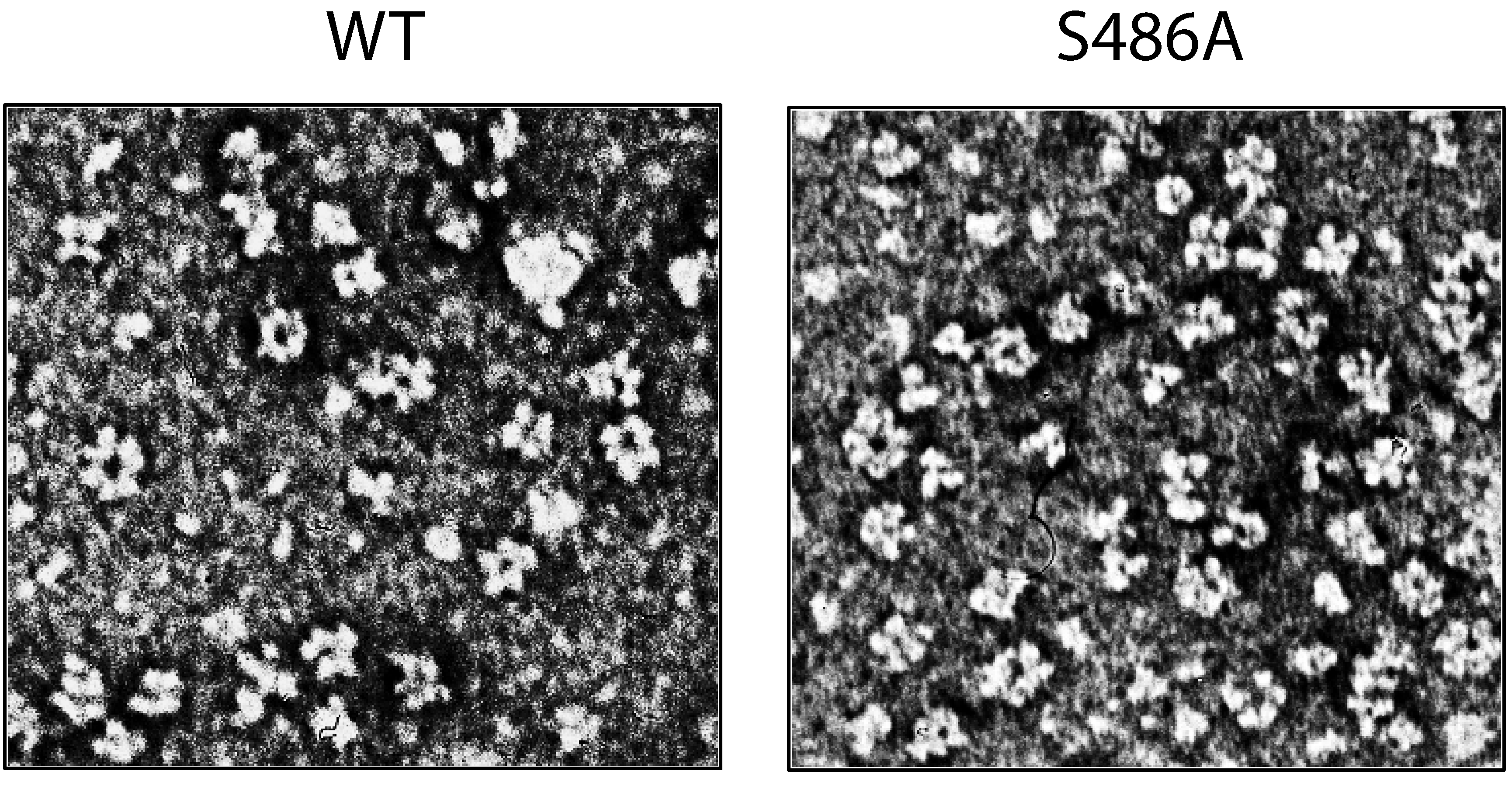

Supplement: S4 Fig — Oligomeric NP was isolated by size exclusion chromatography and immediately prepared for electron microscopy by negative staining with uranyl acetate. Images were taken at 56,000x magnification. Images shown here are the source of the isolated oligomers shown in Fig 4. (TIF) [file ppat.1004826.s009.tif]

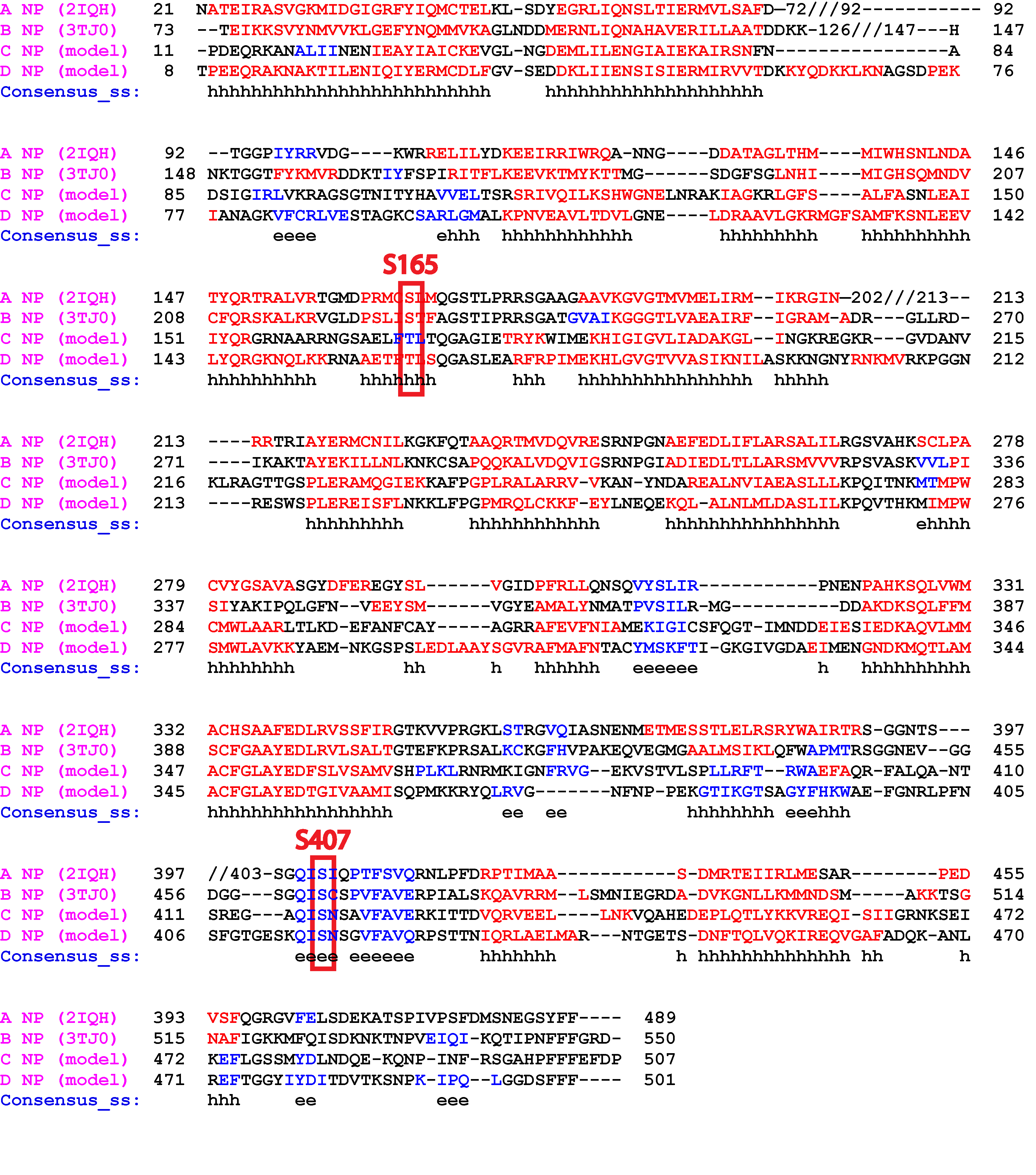

Supplement: S5 Fig — Structures of influenza virus A (A/WSN/1933/H1N1, PDB:2IQH) and B (B/Managua/4577.01/2008, PDB:3TJ0) NP were used as templates for homology modeling of influenza C (C/Ann Arbor/50) and D (D/swine/Oklahoma/1334/2011) using Phyre2. The resultant structure-based alignment is shown. Conserved phosphorylation sites S165 and S407 in influenza A NP align with S226 and S463 in B NP, T169 and S418 in C NP, and T161 and S416 for D NP. Conserved secondary structure helices (h) and beta strands (e) are indicated. Alignment was created with PROMALS3D. Note that sequences derived from these structures do not contain complete N- and C-termini. (TIF) [file ppat.1004826.s010.tif]
